# Genome-wide identification of R2R3-MYB family in wheat and functional characteristics of the abiotic stress responsive gene *TaMYB344*

**DOI:** 10.1186/s12864-020-07175-9

**Published:** 2020-11-12

**Authors:** Qiuhui Wei, Rong Chen, Xin Wei, Yuheng Liu, Shujuan Zhao, Xiaopu Yin, Tian Xie

**Affiliations:** Key Laboratory of Elemene Class Anti-cancer Chinese Medicine of Zhejiang Province, Engineering Laboratory of Development and Application of Traditional Chinese Medicine from Zhejiang Province, Holistic Integrative Pharmacy Institutes, School of Medicine, Hangzhou Normal University, No.2318 Yuhangtang Road, Hangzhou, 311121 People’s Republic of China

**Keywords:** Genome, Wheat, MYB family, Expression pattern, Abiotic stress

## Abstract

**Background:**

MYB superfamily is one of the most abundant families in plants, which plays important roles in plant growth, development, and productivity. However, to date, researches on MYBs in wheat (*Triticum aestivum* L.) are scattered mostly, not comprehensive.

**Results:**

In this study, a total of 393 R2R3-MYBs and 12 R1R2R3-MYBs were identified and analyzed including gene structure, chromosomal distribution, synteny relationship, and evolutionary relationship. Then, 29 clusters tandem duplication and 8 clusters segmental duplication genes were discovered. The expression profile of the identified genes under abiotic and biotic stress was analyzed using RNA-seq data. Based on expression patterns analysis, we screened many candidate genes involved in plant response to abiotic and biotic stress. Among them, the functional characteristics of *TaMYB344* were further studied. TaMYB344 was localized in the nucleus and functioned as a weak transcriptional activator. We demonstrated that *TaMYB344*-overexpressing transgenic tobacco plants had enhanced tolerance to drought, heat, and high salt stress.

**Conclusions:**

In this study, 393 R2R3-MYBs and 12 R1R2R3-MYBs in wheat were systemically identified and analyzed. Differential expression analysis indicated that many R2R3-MYBs were involved in abiotic and biotic stress response. We identified a potential candidate gene *TaMYB344*, overexpression of which in tobacco plants enhanced drought, heat, and salt stress tolerance. These results will provide abundant molecular data for breeding new varieties of wheat in the future.

**Supplementary Information:**

**Supplementary information** accompanies this paper at 10.1186/s12864-020-07175-9.

## Background

The MYB gene family is one of the largest transcription factor (TF) families in plants containing conserved MYB DNA binding domain [1]. MYB DNA binding domain consists of imperfect repeats, R1, R2, and R3, each containing 52 amino acids and forming a very similar folding architecture. Each repeat, containing three well-defined α-helixes, forms a variant of helix-turn-helix motif with three regularly spaced tryptophan (or hydrophobic) residues between the second and third helix. The third helix of R2 and R3 is involved in specific base recognition in the major groove of the DNA to form a stable combinational structure [2]. MYB superfamily can be divided into four subfamilies based on the number of “R” in MYB genes, named R-related (1R), R2R3-MYB (2R), R1R2R3-MYB (3R), and 4R-MYB (four R1/R2-like repeats) respectively. Among them, R1R2R3-MYB is a smaller subfamily typically containing five members in higher plant genomes. R2R3-MYB subfamily, including the most number of MYB genes, arouses researcher’s interest because of its diverse functions involved in primary and secondary metabolism, cell fate and identity, developmental processes, and response to biotic and abiotic stress [2]. In the process of plant evolution, R1R2R3-MYB genes may evolve from R2R3-MYB by acquiring a R1 repeat, or R2R3-MYB genes may evolve from R1R2R3-MYB by losing a R1 repeat [3].

In plants, the first MYB gene *COLORED1*(*C1*) isolated from maize (*Zea mays*), encodes a c-MYB-like transcription factor involved in anthocyanin biosynthesis pathway in the aleurone of maize kernels [4]. With the development of genomics and molecular biotechnology, more and more MYB gene families and functions have been identified and studied in all kinds of plants. The MYB family of Arabidopsis, which is a kind of common model plant, had been early identified [2, 5, 6]. Based on the identification of AtMYBs family, numerous AtMYBs were confirmed to be involved in plant response to biotic and abiotic stress. For example, AtMYB111 plays the role as a positive regulator in salt stress response depending on its regulation on flavonoid synthesis by activating the transcription of chalcone synthase (CHS), flavanone carboxylase (F3H), and flavonol synthase 1 (FLS1) [7]. AtMYB59 negatively regulates Ca^2+^ homeostasis and signaling during Ca^2+^ deficiency, thus controlling plant growth and stress response [8]. Similarly, the MYB family of rice (*Oryza sativa* L.), which is a model plant of monocotyledons, had also been identified [5, 9]. OsMYB30 plays an important role in brown planthopper resistance response [10]. OsMYB6 functions as a stress-responsive factor which plays the role as a positive regulator in response to drought and salt stress resistance [11]. Moreover, the MYB families of other plant species were also identified and studied, such as *Glycine max*, *Vitis vinifera*, *Zea mays*, *Solanum tuberosum*, *Gossypium*, *Medicago sativa*, *Solanum lycopersicum*, and *Brassica napus* etc. [12–19].

However, researches on MYBs in wheat (*Triticum aestivum* L.) are scattered mostly, not comprehensive. As one of the three most important cereals, wheat is widely planted all around the world. The yield and quality of wheat have suffered seriously environmental influences including drought, heat, cold, high-salt, phosphorus starvation, and disease stress etc.. Previous studies indicate that MYB proteins participate in wheat response to environmental stress. For instance, TaMYB can bind to the specific MYB binding sites in the promoter fragments to up-regulate the expression levels of *GAPCp2* and *GAPCp3*, which are involved in drought stress response in wheat [20]. Overexpression of *TaMYBsm3-D* increases the drought tolerance of transgenic Arabidopsis through up-regulating *P5CS1*, *DREB2A*, and *RD29A* [21]. TaMYB31 plays the role as a positive regulator in drought resistance through up-regulating wax biosynthesis genes and drought-responsive genes [22]. Although a few MYB genes have been identified and characterized in wheat, there are still many members of MYB superfamily unknown due to the complex and massive genome of hexaploid wheat.

In this study, we performed genome-wide identification of MYB transcription factors in wheat. R2R3-MYB and R1R2R3-MYB subfamilies were identified and analyzed including gene structure, chromosomal distribution, synteny relationship, and evolutionary relationship. We also analyzed the spatial and temporal expression profiles as well as differential expression profiles of wheat MYB genes under biotic and abiotic stress according to wheat RNA-seq database. Among them, the up-regulated R2R3-MYB gene *TaMYB344* was cloned and studied. The results will provide a comprehensive understanding of MYB family, and lay a theoretical foundation for further exploring the potential roles of MYBs in wheat under adverse environment. All the data will provide molecular database for breeding new varieties of wheat.

## Results

### Identification and characterization analysis of TaMYBs in wheat

To identify the *TaMYBs* in wheat, Hidden Markov model (HMM) profile of the MYB domain (PF00249) queried the hmmsearch program (HMMER3.0 package) against the wheat protein database (IWGSC RefSeq v1.0). After the redundant transcripts were removed, all the candidates were verified via SMART, hmmscan, and NCBI-CD-search. A total of 393 R2R3-MYB genes and 12 R1R2R3-MYB genes were identified, of which the corresponding protein sequences, coding sequences (CDS), and genomic sequences were present in Additional file 1: Table S1. They were named from *TaMYB1* to *TaMYB393* and *TaMYB3R1* to *TaMYB3R12*, ordered by their location in chromosome from the top to bottom, from 1 to 7, and from A, B, to D.

In order to understand the structure of *TaMYB* genes, the intro-exon pattern was analyzed by GSDS 2.0 based on CDS and genomic sequences in Additional file 1: Table S1. The results showed that more than 84% (333) R2R3-MYBs of 393 R2R3-MYBs contain 1–3 introns, while about 14% (54) R2R3-MYBs have no intron. The homologous genes *TaMYB23*, *TaMYB156*, and *TaMYB282* located on 2A, 2B, and 2D chromosomes respectively, have the largest number of 11 introns. The intron numbers of 12 R1R2R3-MYB range from 2 to 11 (Additional file 1: Table S2, Additional file 2: Figure S1–4).

The amino acid sequence length of TaMYBs is in the range of 170–1084 bases. The isoelectric point (pI) and molecularweight (Mw) of TaMYBs range from 4.57 to 11.39, and 19.3 to 121 KDa, respectively. To provide possible clues for function research, the subcelluar location was predicted. The results showed that vast majority of TaMYBs protein were located in the nucleus, only three TaMYBs (TaMYB212, TaMYB293, and TaMYB342) were predicted to be located in mitochondria. All above informations were present in Additional file 1: Table S2.

### Analysis of chromosome distribution, gene duplication, and genome synteny of TaMYBs

**Fig. 1:**
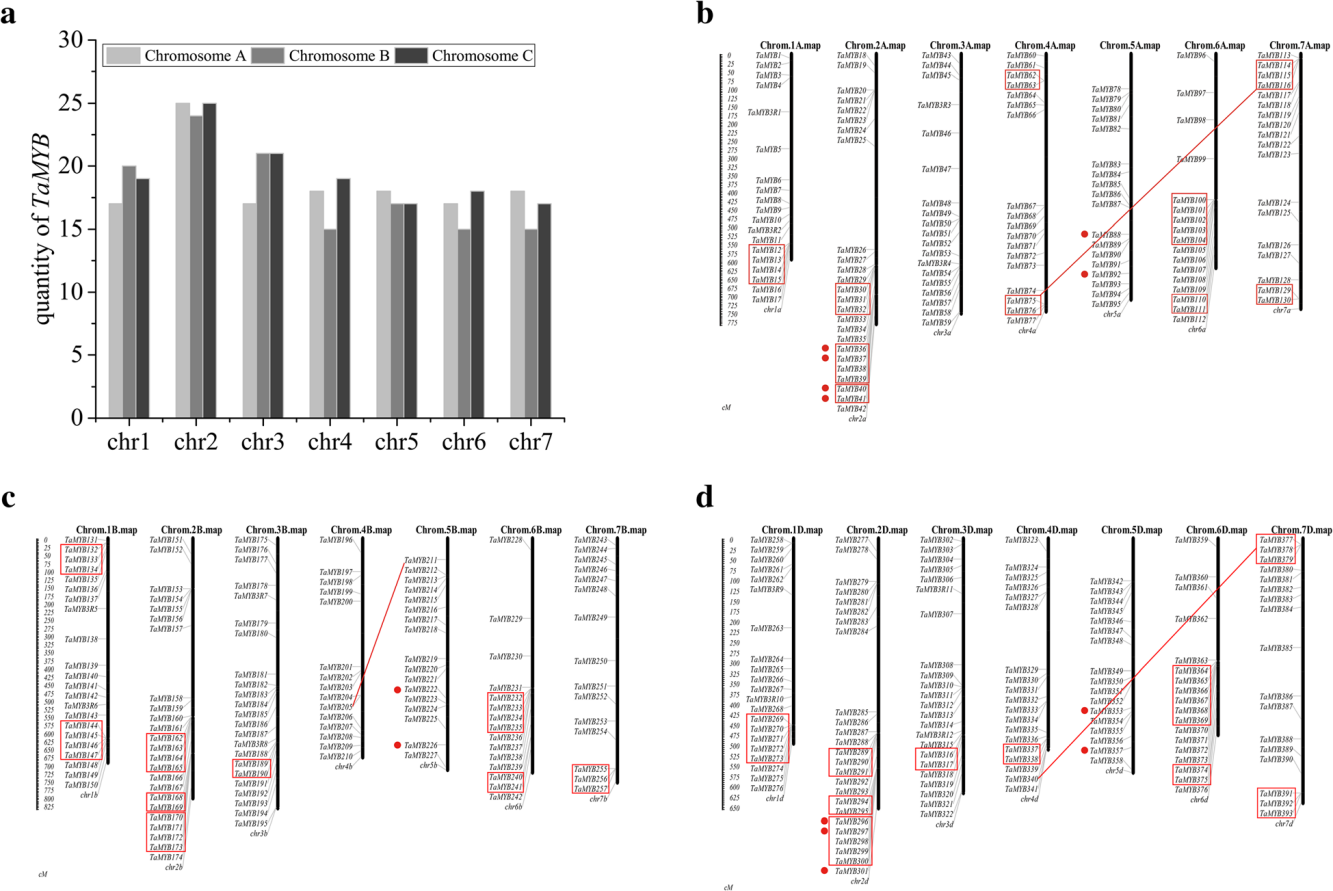
Distribution of 405 Ta*MYBs* on 21 wheat chromosomes. **a** Numbers of *TaMYBs* on each wheat chromosome. **b**-**d** Relative position of *TaMYBs* distribution on 21 wheat chromosomes. The picture was drawn by MapInspect. Tandem duplication genes are marked by red boxes, and segmental duplication genes are marked by red lines and dots

Hexaploid wheat consists of three subgenomes A, B, and D, which respectively contains 7 chromosomes. The position information of *TaMYBs* in chromosomes (Additional file 1: Table S2), was acquired by matching *TaMYBs* CDS with wheat genome database (IWGSC RefSeq v1.0). Then 405 *TaMYBs* were assigned on 21 wheat chromosomes, and the marked position only represented the relative location instead of the real location (Fig. 1). There are 130, 127, and 136 *TaMYBs* respectively located on genome A, genome B, and genome D. Chromosomes 2A/B/D contain the maximum number of *TaMYBs*, while 4B, 6B, and 7B have the minimum number of *TaMYBs* (Fig. 1a). The *TaMYBs* mainly distributed in bottom of chromosomes, while relative high density of *TaMYBs* was found on the top of 7A/B/D chromosomes (Fig. 1b-d).

**Fig. 2:**
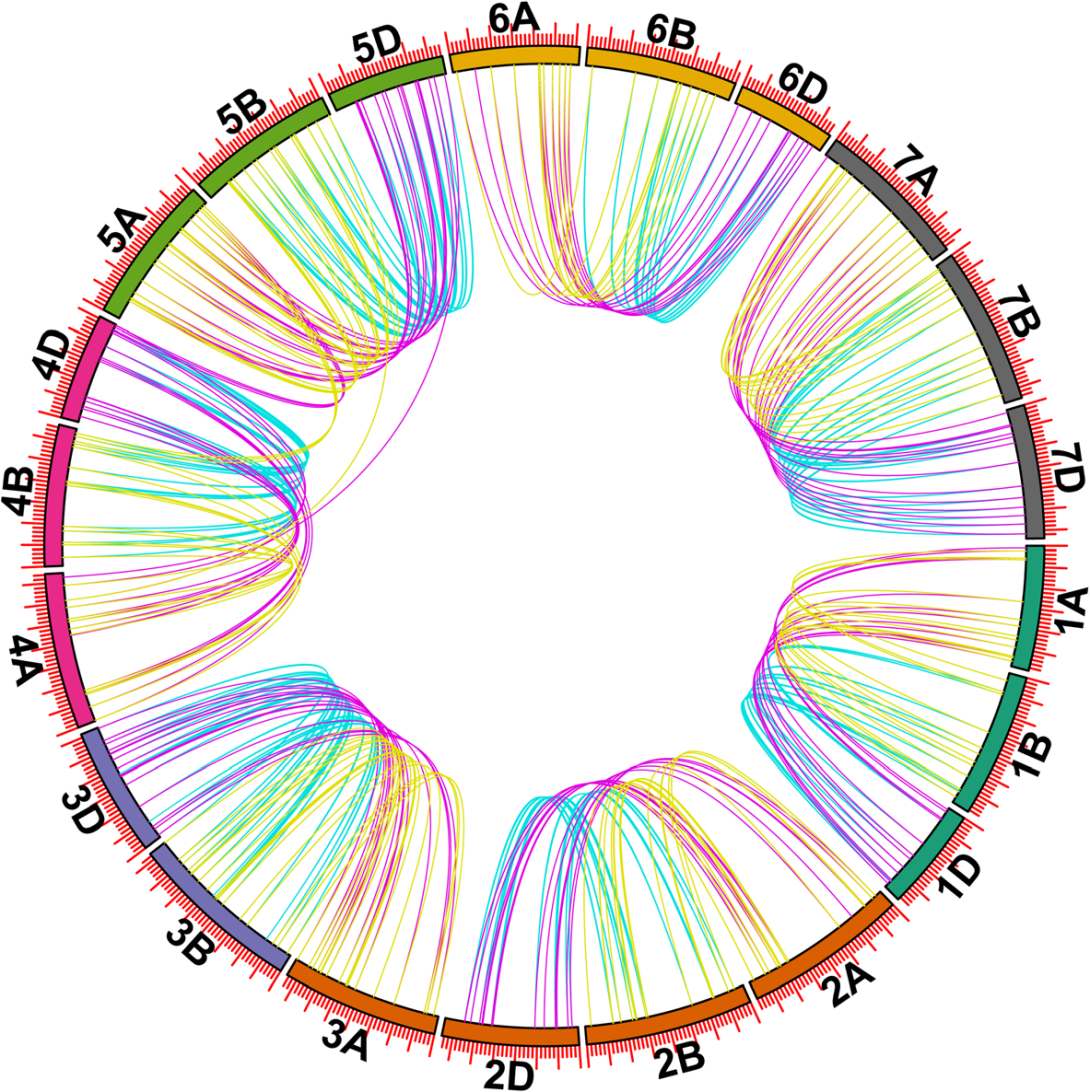
Syntenic relationship of *TaR2R3-MYBs* in wheat genome A/B/D. The positions of all the *TaR2R3-MYBs* are depicted in the three subgenomes of wheat. The different color lines indicate the synteny of *TaR2R3-MYBs* among genome A/B/D. The picture was drawn with TB tools

Tandem gene duplication and segmental gene duplication are important factors in biological evolution for naturally new genes generation and gene-family expansion [23]. In our study, 29 clusters tandem duplication genes and 8 clusters segmental duplication genes were discovered in TaMYB family of wheat (Fig. 1**)**. In order to understand the selection mode of duplicated *TaMYBs* in wheat, Ka/Ks values were calculated. All of the duplication gene pairs showed Ka/Ks < 1 (Additional file 1: Table S4), indicating that *TaMYBs* underwent the purity selection in wheat. In addition, *TaMYBs* were identified to involve in chromosome translocation and inversion in subgenome A (Additional file 1: Table S3). Thirteen *TaMYBs* from *TaMYB60* to *TaMYB72* were involved in pericentric inversion between short and long arms of chromosome 4A. The reciprocal translocation of *TaMYB73* with *TaMYB93/94/95* happened between the long arms of chromosome 4A and 5A. The reciprocal translocation of *TaMYB26* with *TaMYB27* and *TaMYB105* with *TaMYB100/101/102/103/104*, occurred respectively in chromosome 2A and 6A. The syntenic relationships of *TaMYBs* between subgenome A, subgenome B, and subgenome D in wheat were analyzed and presented in Fig. 2 and Additional file 1: Table S3. There are more than 80% of TaMYBs having all the three homologs across subgenome A, B, and D.

### Phylogenetic analysis of the R2R3-MYBs

**Fig. 3:**
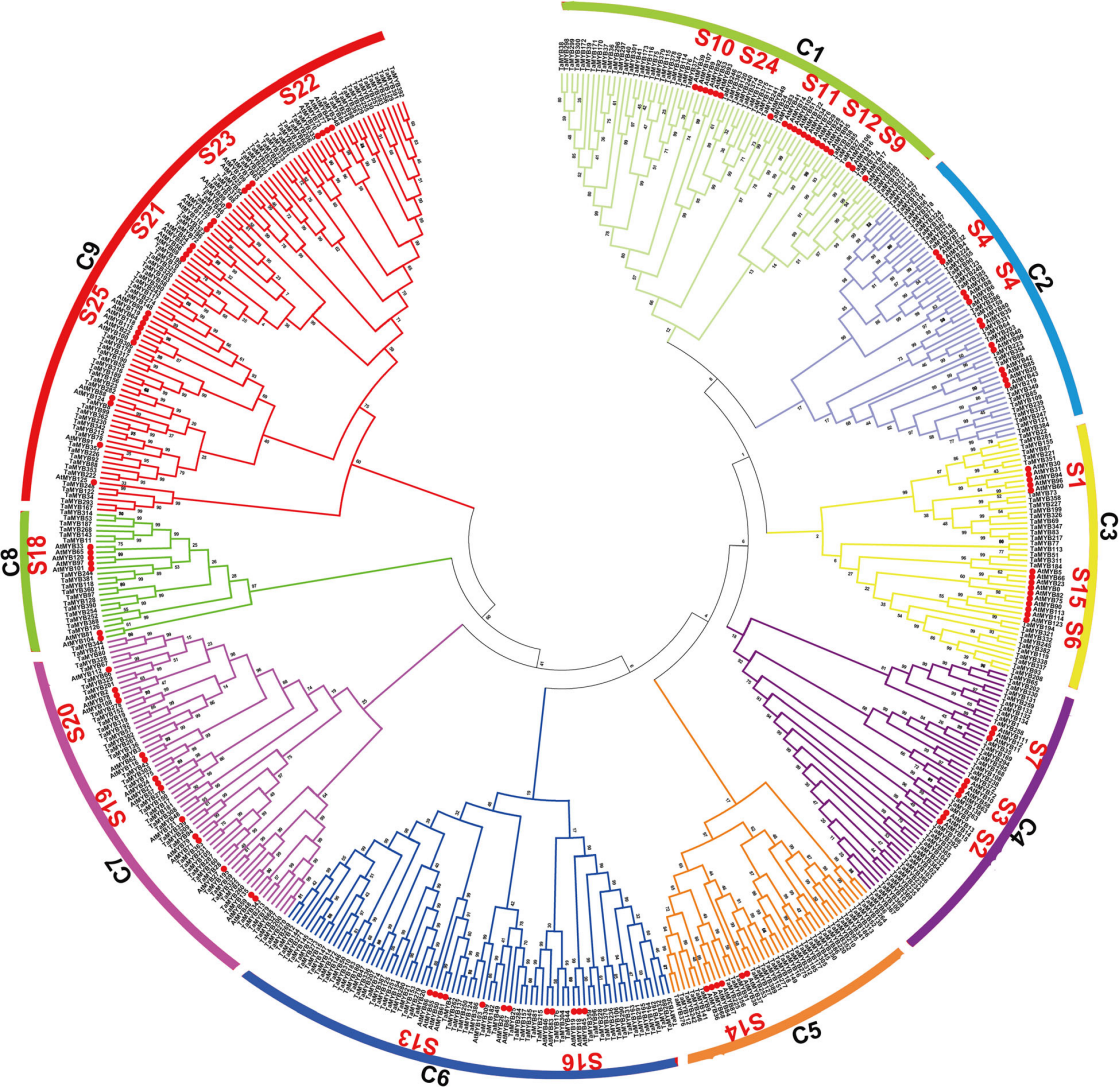
Phylogenetic tree of R2R3-MYBs in wheat and Arabidopsis. The sequences contain 393 TaR2R3-MYBs in wheat and 126 AtR2R3-MYBs in Arabidopsis. All R2R3-MYBs were divided into 9 clusters (C1-C9). S1-S25 represent the previous functional classification of 126 AtR2R3-MYBs in Arabidopsis [2]. The picture was generated by using MEGA 7 software coupled with Neighbor-Joining method with a bootstrap of 1000 replicates

The evolutionary relationship of identified 393 R2R3-MYBs was analyzed by MEGA 7.0. All R2R3-MYBs were classed into 10 clusters (C1-C10) each containing the number of 82, 49, 54, 64, 48, 3, 22, 55, 4, and 12 R2R3-MYBs (Additional file 2: Figure S5). The homologous genes TaMYB6/TaMYB139/TaMYB264 respectively located in subgenome A//B/D were independent in C6. TaMYB8 and homologous genes TaMYB99/TaMYB230/TaMYB362 were independent in C9. In addition, the phylogenetic tree of identified 393 R2R3-MYBs in wheat with 126 R2R3-MYBs in Arabidopsis was constructed. As shown in Fig. 3, all the R2R3-MYBs were classed into 9 clades, and each clade contained R2R3-MYBs from wheat and Arabidopsis. The result suggested the existence of a common ancestor before the divergence of monocots and dicots. Meanwhile, the further subdivision of phylogenetic relationship of TaMYBs with AtMYBs which have the clearest protein classification and function research [2], will provide more useful references for function identification in wheat.

### Expression profiles of *TaMYB* genes in different tissues

**Fig. 4:**
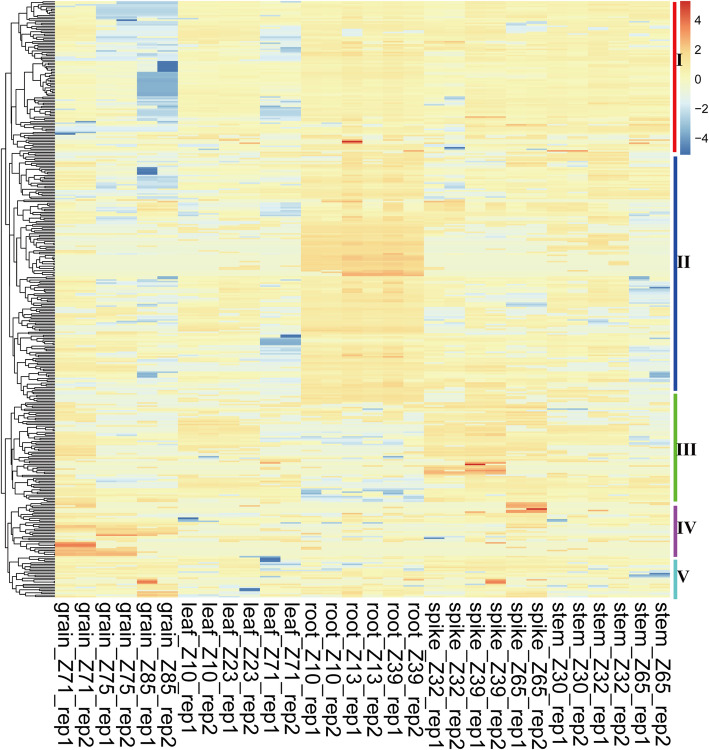
Expression profiles of *TaR2R3-MYB* genes in different tissues. Row coordinate meanings are as following: Grain_Z71, _Z75 and _Z85: grains at 2, 15, and 30 days post anthesis (dpa) stages, respectively; Leaf_Z10, _Z23, and _Z71: leaf at seedling stage, flag leaf at tillering stage, and leaf at 2 dpa stage; Root_Z10, _Z13, and _Z39: roots at seedling, three leaf, and flag leaf stages, respectively; Spike_Z32, _Z39, and _Z65: spikes at two-node, flag leaf, and anthesis stages, respectively; Stem_Z30, _Z32, and _Z65: stems at 1 cm spike, two-node, and anthesis stages, respectively. Heatmap was created by R program based on the data of the transcript per million (TPM) values which were normalized with Z-score method. The red and blue cells respectively represent highest and lowest expression level

There are great difficulties to research the biological function of TaMYBs, because MYB family is one of the largest families, and the members perform a variety of functions in plants [2]. As we all know, the biological roles have a certain degree of correlation with their expression patterns in plants. To analyze the expression patterns of 405 identified *TaMYBs*, public RNA-seq data containing 15 different growth stages of root, stem, leaf, spike, and grain throughout the life cycle of the wheat was obtained from expVIP website (Additional file 1: Table S5). Hierarchical cluster analysis was performed based on the transcript per million (TPM) values of 405 TaMYBs (Fig. 4). The expression level of five R2R3-MYB members (*TaMYB1*, *TaMYB148*, *TaMYB302*, *TaMYB332*, and *TaMYB339*) was not detected, which maybe specially expressed in other tissues than the mentioned 15 tissues or under certain special conditions. Among them, homologous genes *TaMYB178/306* specially expressed in grains at 2 days post anthesis, and tandem duplication gene-pairs *TaMYB296/300* and *TaMYB220* specially expressed in roots of wheat. Other *TaMYBs* expressed in 15 different tissues having a large range of expression level. The expression profiles of *R2R3MYBs* were clustered into 5 groups based on their expression characteristics. In group I, the expression level of *R2R3-MYBs* was significant lower in grains at 15/30 days post anthesis (dpa) and leaf at 2 dpa stages than that in other tissues. Contrary to higher expression level of *R2R3-MYBs* in root in group II, that was lower in root but higher in spikes in group III. In group IV, the expression level of *R2R3-MYBs* was relatively higher in grains than that in other tissues. There is no visible difference of expression patterns in group V. In addition, the tissue-expression profiles of 12 *R1R2R3-MYBs* were also analyzed and exhibited in Additional file 2: Figure S6.

### Expression profiles of *TaMYB* genes under different stress treatment

**Fig. 5:**
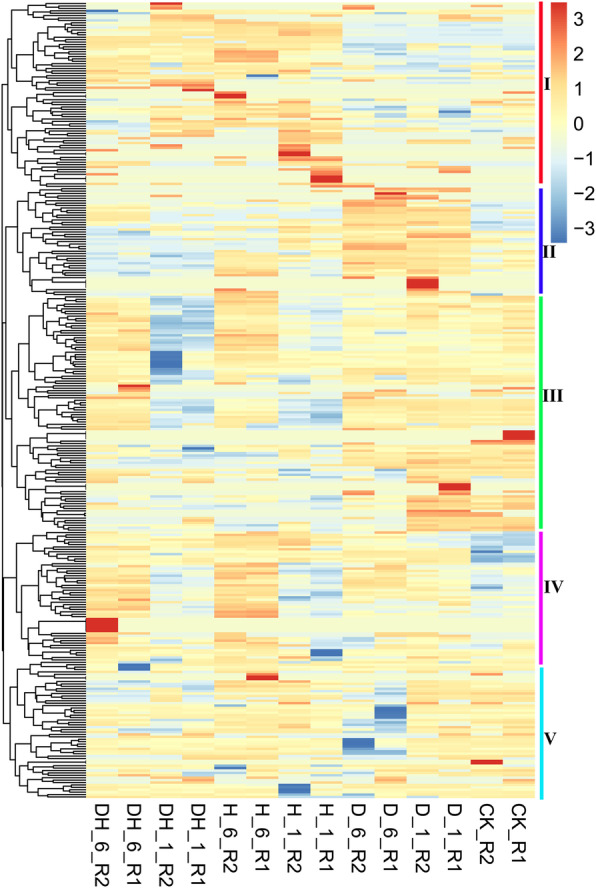
Expression profiles of *TaR2R3-MYB* genes under drought/heat treatment. Row coordinate meaning are as follows: DH_1 and DH_6: drought and heat stress at 1 and 6 h post stress (hps), respectively; H_1, and H_6: heat stress at 1 and 6 hps, respectively; D_1 and D_6: drought stress at 1 and 6 hps, respectively. Heatmap was created by R program based on the data of the transcript per million (TPM) values which were normalized with Z-score method. The red and blue cells respectively represent highest and lowest expression level

Plant growth is often affected by abiotic and biotic stress. Therefore, the expression profiles of identified *TaMYBs* were further analyzed under different stress to clarify their biological function. The RNA-seq data was obtained from expVIP website containing abiotic (drought and heat, cold, and phosphate starvation) and biotic (stripe rust and powdery mildew) stress treatment (Additional file 1: Table S5). Under drought/heat stress, hierarchical cluster analysis was performed based on TPM values of *R2R3-MYBs* in wheat (Fig. 5). Several genes with no detected expression (TPM = 0) were deleted in the heatmap. The result showed that the expression profiles of most of genes in group I, II, and IV were up-regulated. In group I, the expression level of *R2R3-MYBs* was induced and up-regulated by heat (H) stress, and drought plus heat (DH) stress after 1 and 6 h, while those were not induced after drought (D) stress. Instead, the expression level of *R2R3-MYBs* only increased after drought stress in group II with no significant change after H and DH stress. In group IV, the expression level of *R2R3-MYB*s was up-regulated under drought, heat, and DH stress. In addition, expression level of most genes was down-regulated after heat and DH treatment in group III, and expression level of most genes was down-regulated under drought stress in group V. Finally, the up- and down- regulated *R2R3-MYBs* involved in various stress were screened with strict screening conditions (Table 1, Additional file 1: Table S6, Additional file 2: Figure S7–9). The results showed that more *R2R3-MYBs* in wheat involved in drought/heat/cold stress, while less participated in phosphorus (Pi) -starvation/disease stress. In addition, the expression profiles of 12 *R1R2R3-MYBs* under different stress were also analyzed and exhibited in Additional file 2: Figure S6.

**Table 1:**
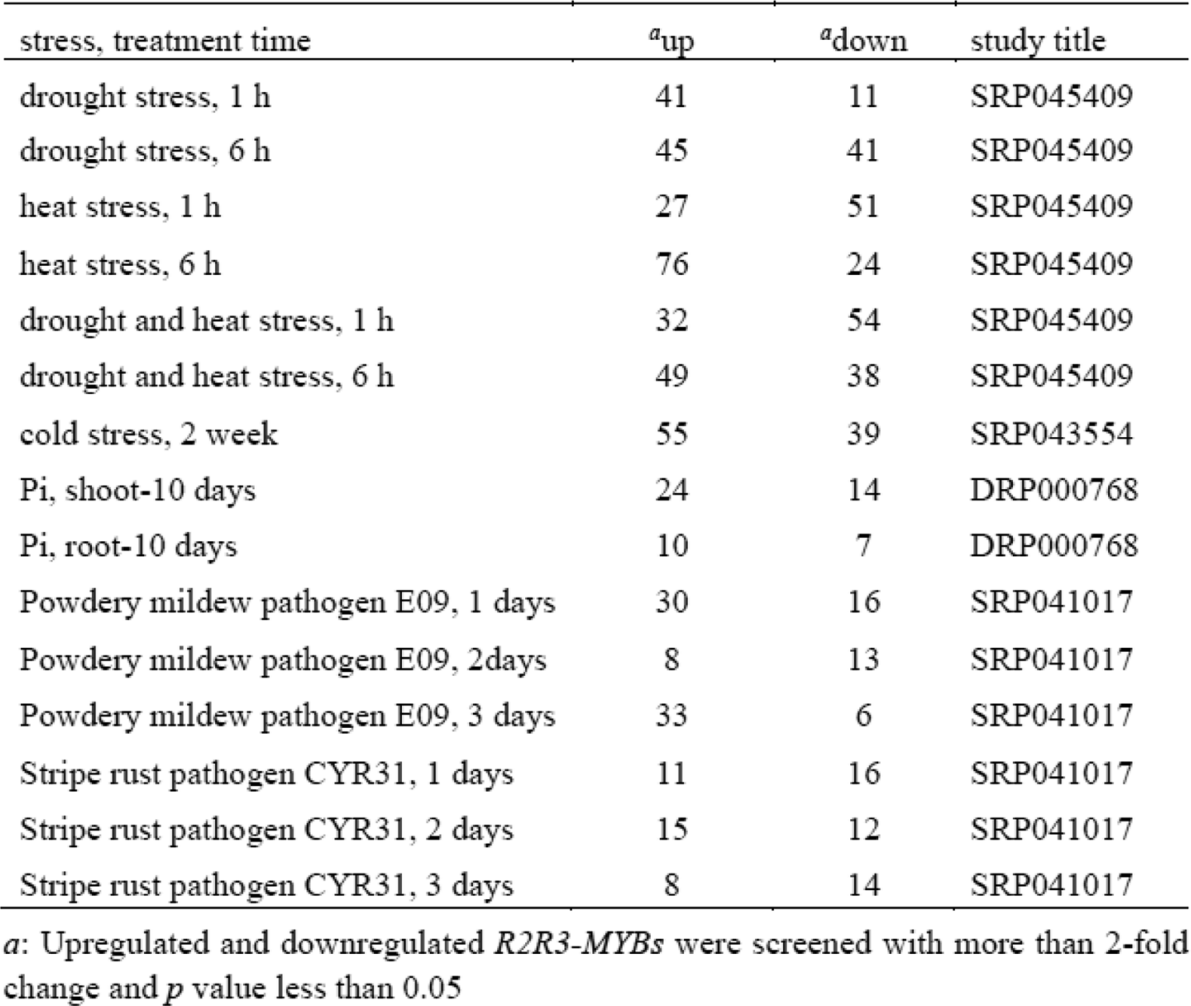
Number of stress-responsive *R2R3-MYB*s in wheat under various stress

**Fig. 6:**
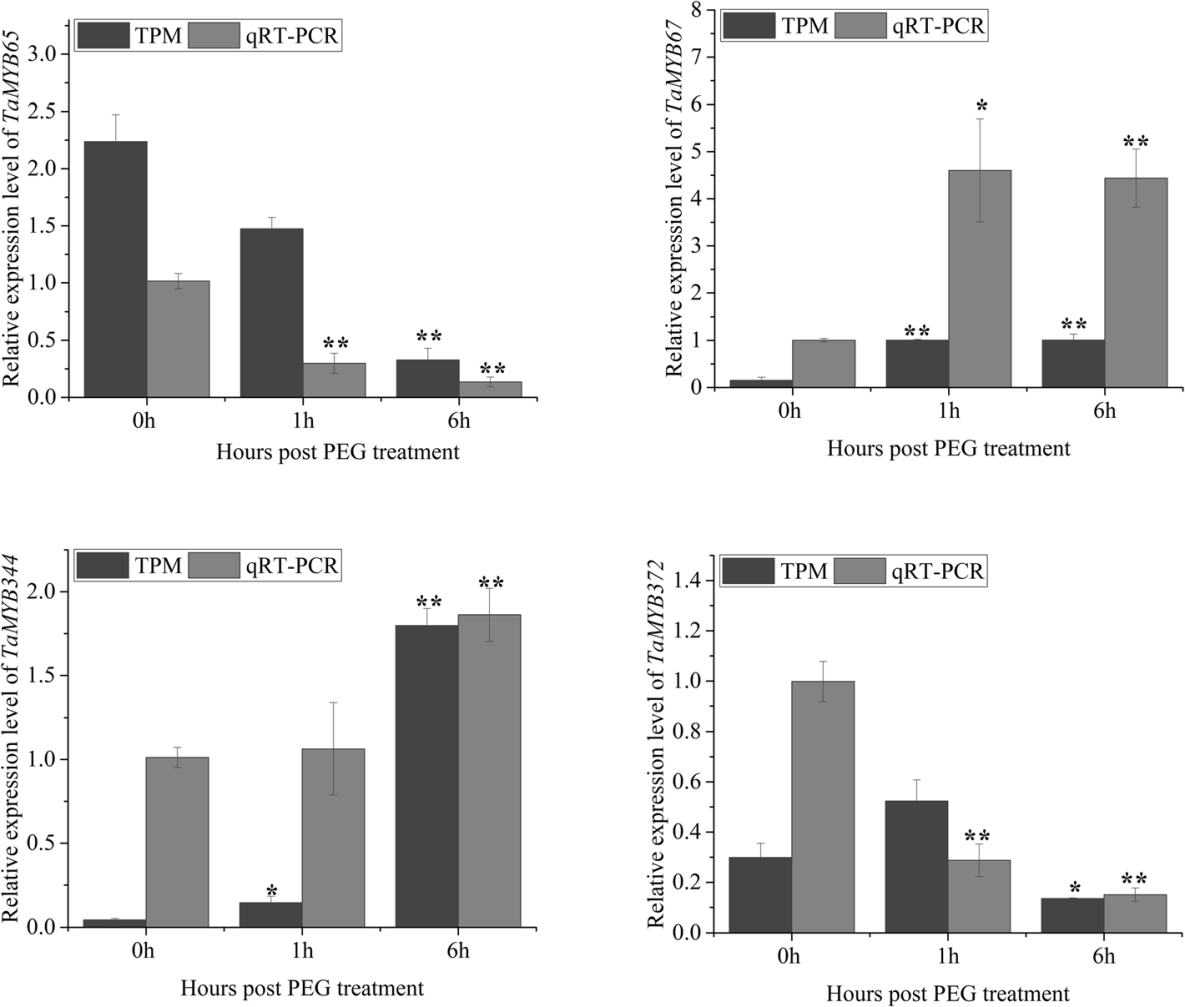
Expression profiles of four selected *TaMYB* genes under drought stress. Dark gray columns represent the transcript per million (TPM) values from RNA-seq data, which reflect the expression level; Gray columns represent the result of qRT-PCR experiments. Asterisks indicate significant difference (^*^P < 0.05; ^**^P < 0.01)

In order to validate the expression profiles of *TaMYBs* calculated based on RNA-seq data, four *TaMYBs* were randomly selected and then determined by qRT-PCR under drought stress treatment (Fig. 6). The experimental results roughly agree with the mentioned results, which indicates the analysis results are reliable.

### Functional characterization of TaMYB344 in transgenic tobacco plants under different abiotic stress

**Fig. 7:**
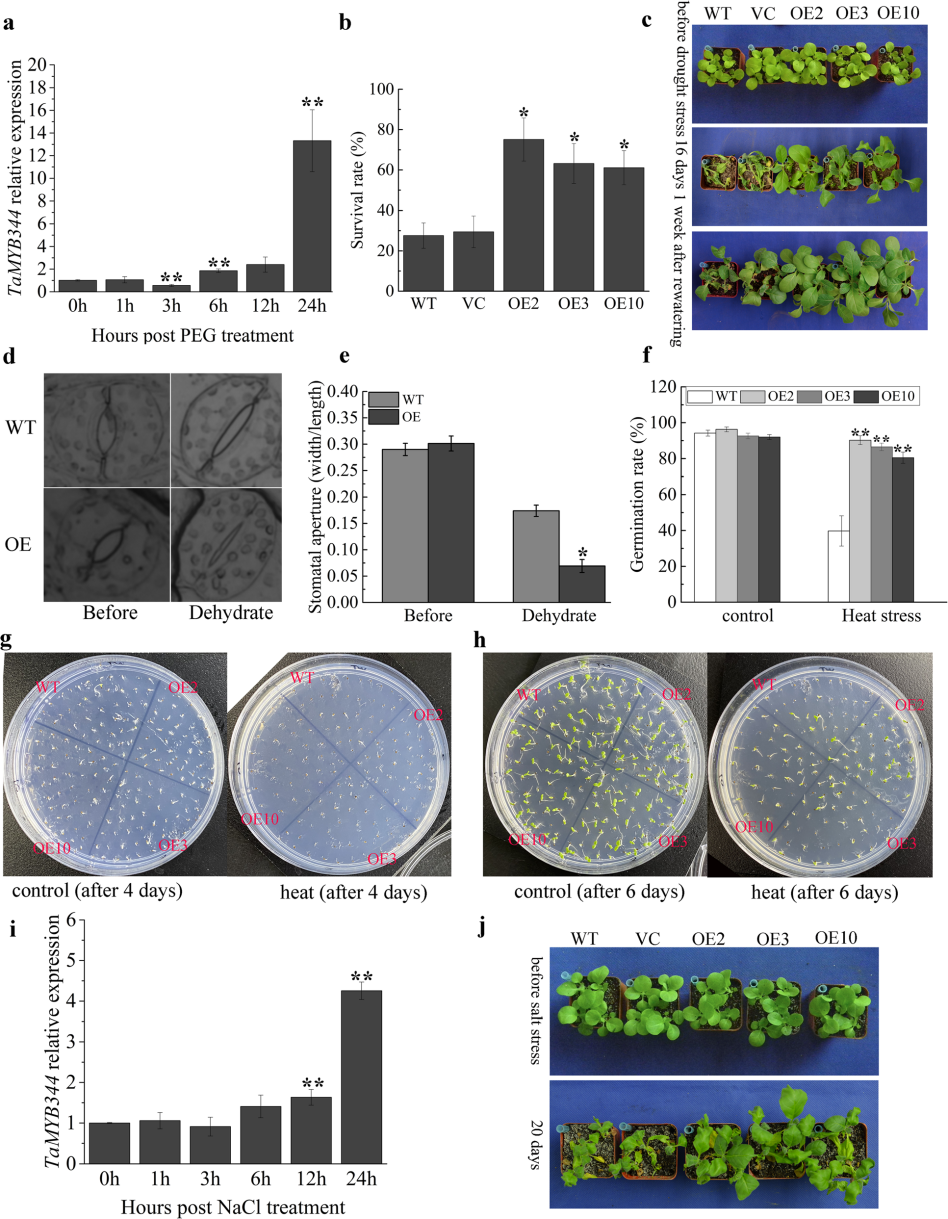
Functional characterization of TaMYB344 in transgenic tobacco plants under different abiotic stress. **a** Expression patterns of *TaMYB344* in 14-day-old wheat seedlings after treatment with 20% PEG6000. **b**, **c** Phenotypes and survival rates of WT, VC, and OE lines (OE2, OE3, and OE10) after drought treatment. **d** Stomatal aperture after dehydration treatment. **e** Width/length ratios of stomata. **f** Germination ratios of WT and OE lines after heat treatment. **g**, **h** Phenotype of WT and OE lines after heat treatment. **i** Expression patterns of *TaMYB344* in 14-day-old wheat seedlings after treatment with 200 mM NaCl. **j** Phenotype of WT, VC, and OE lines after salt treatment. At least three independent biological replicates were performed. Vertical bars refer to ±SE (*n* = 3). Asterisks indicate significant difference (^*^P < 0.05; ^**^P < 0.01)

In the study, quantitative real-time PCR (qRT-PCR) was used to validate *TaMYB344* expression pattern under different abiotic stress. *TaMYB344* expression level was fluctuating within 12 h, and finally sharply increased about 13-fold after 24 h under 20% PEG6000 treatment (Fig. 7a). The expression level of *TaMYB344* responding to salt stress was also detected. Similarly, *TaMYB344* expression level remained relatively stable within 6 h, and gradually increased at 12 h, then quickly increased about 5-fold after 24 h under treatment with 200 mM NaCl (Fig. 7i). Meanwhile, the 1500 bp upstream sequence as the promoter of *TaMYB344* were analyzed. Responsive elements HSE, MBS, ABRE, TCA, and TGA were discovered (Additional file 1: Table S7). The MBS and HSE *cis*-elements had been studied to respectively involve in drought and heat stress response. The results can provide some clues for the biological function studies.

To further investigate the function of TaMYB344 in abiotic stress tolerance, three transgenic tobacco plants highly overexpressing *TaMYB344* (OE2, OE3, OE10) were obtained (Additional file 2: Figure S10). Under normal conditions, wild type (WT), vacant control (VC), and OE lines showed similar germination rates and phenotypes. For drought tolerance analysis, water was withheld from 4-week-old plants in soil for 16 days. Then WT and VC lines became seriously wilted and dead. By contrast, the transgenic lines OE2, OE3, and OE10 showed only few deaths (Fig. 7c). After re-watering for 1 week, the survival rate of the transgenic plants was 60–80%, which was obviously higher than those of WT and VC (< 30%) (Fig. 7b). In our study, the status of stomatal closure was observed and the stomatal width:length ratio was measured under dehydration treatment. Results showed that the stomatal aperture of OE lines was smaller than that of the WT (Fig. 7d, e). For high temperature tolerance analysis, tobacco seeds of WT, OE2, OE3, and OE10 after surface-sterilizing were treated in dark plant incubator at 50 °C, for 1 h. Then, all seeds were sown on 1/2 Murashige and Skoog (MS) medium and incubated in the growth chamber (12 h light/12 h dark cycle at 22 °C). Meanwhile, the tobacco seeds without heat stress were also incubated in the same environment as control. After 4 days, the germination rates of OE lines (about 80%) were significantly higher than that of WT (about 40%) **(**Fig. 7f), and the phenotypes were photographed after incubation for 4 and 6 days (Fig. 7g, h). For salt tolerance analysis, 4-week-old plants were irrigated with 500 mM NaCl solution for 20 days. The WT and VC lines were serious wilted and dead, whereas the transgenic plants became relatively light wilting (Fig. 7j). All above results affirmed that TaMYB344 positively regulated plants stress tolerance to drought, heat, and salt stress.

### Subcellular localization and transcriptional activation analysis of TaMYB344

**Fig. 8:**
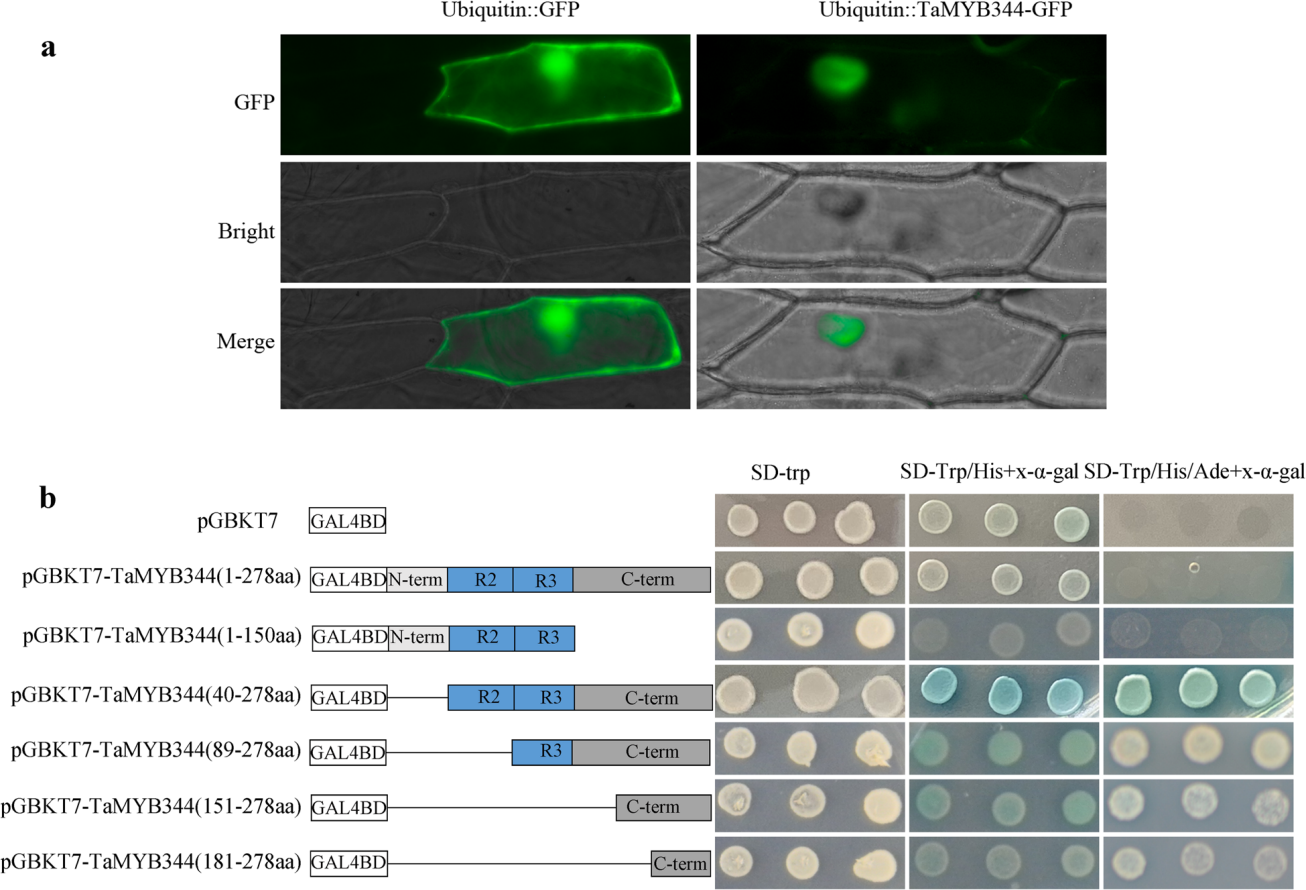
Subcellular localization and transcriptional activity analysis of TaMYB344. **a** Subcellular localization of TaMYB344. Recombinant ubiqutin:: TaMYB344-GFP and control vector ubiquitin::GFP were respectively transformed into onion epidermal cells and observed with fluorescence microscopy. **b** Transactivation activity of TaMYB344 in yeast. Schematic diagrams illustrate the different portions of TaMYB344 ORF. Recombinant were transformed into yeast strain AH109, and the transformants were screened by SD/−Trp, SD/−Trp/−His+X-a-gal, and SD/−Trp/−His/−Ade + X-a-gal media. At least three independent biological replicates were performed

In order to further understand the functional mechanism of TaMYB344, the localization of TaMYB344 was analyzed in onion epidermal cells by transient expression experiment. The vector expressing the fused TaMYB344-GFP protein under the control of a maize ubiquitin promoter was constructed. Transient expression result showed that the fluorescence of TaMYB344-GFP was exclusively localized in the nucleus, whereas that of the control GFP protein was diffused throughout the cell (Fig. 8a). These results suggested that TaMYB344 is a nuclear-localized protein.

The transactivation activity of TaMYB344 was verified in yeast. The complete and various truncated *TaMYB344* open reading frames (ORFs) were cloned into pGBKT7 plasmid to obtain GAL4BD-*TaMYB344* recombinants. The recombinants were respectively transformed into the yeast strain AH109 to examine the transactivation ability of TaMYB344. All the transformants and the negative control pGBKT7 grew well on SD/−Trp medium. Truncated TaMYB344 containing N-terminal did not grew on SD-Trp/His or SD-Trp/His/Ade medium with X-α-galactoside (X-α-gal). Transformants containing the TaMYB344 C-terminal grew well and turned blue on SD-Trp/His medium with X-α-gal. On SD-Trp/His/Ade medium, only truncated TaMYB344 (40–278 aa) transformants grew well and turned blue, while others grew without turning blue. Surprisingly, the growth status of transformants containing complete ORF of TaMYB344 grew on SD-Trp/His or SD-Trp/His/Ade medium with X-α-gal was not stable. In order to further confirm the results, one hundred repeats were performed. The results showed that all repeats grew well on SD-Trp/His medium with X-α-gal, while only 33% turned blue; meanwhile only 12% repeats grew well and turned blue on SD-Trp/His/Ade medium with X-α-gal while others cannot grow (Fig. 8b). All these results suggested that the complete TaMYB344 has relatively weak activity, and truncated TaMYB344 missing N-terminal (1 ~ 39 aa) has relatively strong transactivation activity.

## Discussion

### R2R3-MYB gene family in wheat

The MYB gene family is one of the largest families in plants. To date, several R2R3-MYB families have been identified and analyzed, such as three model plants *Arabidopsis thaliana* (126) [6], *Oryza sativa* (102) [9], and *Brachypodium distachyon* (85) [24], as well as *Zea mays* (157) [19] and *Phyllostachys edulis* (82) [25]. The wheat genome has been sequenced, yet the R2R3-MYB transcription factors have not been completely identified, excluding a rough analysis of 23 isolated wheat R2R3-MYB genes [26]. In this study, we systematically identified 130, 127, and 136 R2R3-MYBs in subgenome A/B/D of hexaploid wheat, respectively (Additional file 1: Table S1–3). The number of R2R3-MYBs in each wheat subgenome A/B/D (each like a diploid) is more than that of diploid model plants *Arabidopsis thaliana*, *Oryza sativa*, and *Brachypodium distachyon*. It indicates that the R2R3-MYB family in wheat has expanded along with genome duplication. Duplication, translocation, and inversion are closely related to plant evolution. In our study, 29 clusters of tandem duplications and 8 clusters segmental duplications (Fig. 1) of R2R3-MYBs were identified in wheat. The duplication events of MYB family also occurred in other plants, such as *Medicago truncatula*, *Solanum tuberosum*, *Brassica napus*, *Gossypium raimondii* etc. [16, 18, 27, 28]. Most homologous chromosomes of hexaploid wheat (AABBDD) are collinear, while chromosomes 4A and 5A happened reciprocal translocations and pericentric inversion in the process of evolution [29]. In our study, thirteen TaMYBs from *TaMYB60* to *TaMYB72* were pericentric inversion in chromosome 4A; reciprocal translocation of *TaMYB73* with *TaMYB93/94/95* happened between chromosome 4A and 5A (Additional file 1: Table S3). These are similar with TaPP2C family in wheat, inversion and translocation events of which also occurred in chromosome 4A and 5A [30]. Moreover, the reciprocal translocations were also discovered between chromosome 2A and 6A.

### The phylogenetic analysis and expression profiles

To understand the evolutionary relationship of MYB genes, we constructed the phylogenetic tree with MYBs from wheat and *Arabidopsis thaliana* (Fig. 3). All MYBs from wheat were classified into nine different classes with MYBs from Arabidopsis, according with that the MYB family is relatively conservative between different plants in evolution [18]. The conservation of gene structure often leads to conservation of gene function. R2R3-MYBs in Arabidopsis were divided into different subgroups (S1-S25), the members of which had close relationships and similar biological functions [2, 6]. For example, AtMYB7/4/32, the members of subgroup 4 in Arabidopsis, encode transcriptional repressors and participate in secondary metabolic regulation [31–33]. Therefore, the homologous TaMYBs of AtMYB7/4/32 (Class 2, Subgroup4) in wheat may be putative repressors participating in secondary metabolic regulation. The TaMyb1D (named TaMYB355 in our study) belonging to subgroup 4 was reported to function as a negative regulator of phenylpropanoid metabolism [34]. Similarly, TaMYB4 (named TaMYB385 in our study), the member of subgroup 4, negatively regulates the lignin biosynthesis in wheat [35]. Besides, many MYBs were reported to be involved in biotic and abiotic stress response in Arabidopsis, wheat, and other plants. In our study, we analyzed the transcriptional profiles of *TaMYBs* under various biotic and abiotic stress in silico (Fig. 5, Table 1, Additional file 1: Table S5–6, Additional file 2: Figure S7–9). Combining evolutionary relationships and expression profiles analysis, the biological functions of TaMYBs can be predicted. For instance, TaMYB232 (belonging to Subgroup2, Class 4) is homologous with AtMYB13/15 which are involved in response to abiotic stress (Fig. 3) [36, 37]. Meanwhile, the expression profiles of TaMYB232 were up-regulated under heat and disease stress (Fig. 5, Additional file 1: Table S6). Therefore, TaMYB232 may be a putative regulator involved in abiotic and biotic stress. The result is consistent with previous study of TaMYB4 (i.e. TaMYB232 in our study) [38]. In class 1, TaMYB24/283 may be involved in biotic and abiotic stress response due to the closely evolutionary relationship with stress response regulation factors AtMYB41/74/102 (subgroup 11) in Arabidopsis [39–41]. The expression levels of TaMYB24/283 were induced by drought and/or heat stress, cold stress, and Pi- starvation in wheat (Fig. 5, Additional file 1: Table S6). TaMYB80 (i.e. TaMYB24 in our study) has been reported to enhance the drought and heat tolerance in Arabidopsis, which is similar with our prediction of gene function [42]. AtMYB2/62/108/112, the members of subgroup 20, are involved in various abiotic stress response by different pathway [43–46]. Twenty-one TaMYBs were also classified into subgroup 20 because of the close relationships with AtMYB2/62/108/112. Among them, the expression profiles of TaMYB67 and TaMYB344 were up-regulated under drought and/or heat stress in silico (Fig. 5, Additional file 1: Table S6) and qRT-PCR analysis (Fig. 6). Therefore, TaMYB344 as well as other members of subgroup 20 may be the regulators of abiotic stress response. In a word, phylogenetic and expression analysis provided effective references for functional research of TaMYBs in wheat.

### TaMYB344 enhances drought, heat, and salt stress tolerance

In our study, *TaMYB344-*overexpressing tobacco plants showed enhanced drought, heat, and salt stress tolerance (Fig. 7). Transpiration is one of the basic physiological activities of plant leaf through stoma, via which more than 95% of water losses in plants [47]. Stomatal closure was more sensitive to dehydration in *TaMYB344* transgenic tobacco plants than that in WT plants under drought stress. The result suggested that TaMYB344 might regulate stomatal closure for adaption to adversely environmental condition. In previous studies, AtMYB44/60, GhMYB5, and many other MYBs in plants also had been identified to regulate stomatal closure to resist drought stress [48–51].

As a transcription factor, TaMYB344 should directly or indirectly regulate the transcription of downstream genes. Hence, the transactivation activity of TaMYB344 is worth noticing. The transactivation activity of complete ORF of TaMYB344 in yeast is weak and not stable (Fig. 8). The result is coincident with that of TaMYB79, which is located in chromosome 5AS and homologous to our reported gene TaMYB344 in chromosome 5DS [42]. However, truncated TaMYB344 missing N-terminal (1 ~ 39 aa) has relatively strong transactivation activity in yeast (Fig. 8). The result is similar with that of transcription factor TaWRKY44. The complete ORF of TaWRKY44 has no transactivation activity while truncated TaWRKY44 missing C-terminal has strong activity [52]. Although the transactivation activity of TaMYB79/TaMYB80/TaWRKY44 was weak even none in yeast, they still played positive roles in plants to resist abiotic stress [42, 52]. Therefore, we speculate that transcriptional activation function of TaMYB344 in plants depends on interaction or modification of other factors. Besides, the expression profiles of *TaMYB344*, which were induced significantly after 24 h, were different with many other genes that were early up- or down- regulated to respond abiotic stress [53, 54]. The above two evidences implied that TaMYB344 might be in the downstream of regulatory network to respond abiotic stress. More detailed transcriptional regulation mechanism of TaMYB344 in wheat will be researched in our future work.

## Conclusions

In conclusion, a total of 393 R2R3-MYB genes and 12 R1R2R3-MYB genes were identified in wheat genome. The gene structure, protein physicochemical properties, chromosome distribution, gene duplication, evolutionary relationship, and expression patterns in different tissues as well as various biotic and abiotic stress were comprehensively analyzed. We also identified a potential candidate gene *TaMYB344*, overexpression of which in tobacco plants enhanced drought, heat, and salt stress tolerances. The target genes or proteins of *TaMYB344* will be further researched to elucidate the mechanism of *TaMYB344*-mediated stress tolerance in our future work. These results in this study will lay a foundation for the future investigation of more potential MYB genes in wheat, then provide abundant molecular data for breeding new varieties of wheat in the future.

## Methods

### Identification of MYB family

The database (IWGSC RefSeqv1.0) of whole genome sequences and protein sequences of wheat was downloaded from URGI (https://wheat-urgi.versailles.inra.fr/Seq-Repository/Assemblies). The HMM profile of the MYB domain (PF00249) was downloaded from Pfam website (http://pfam.xfam.org/family/PF00249). All of the TaMYBs proteins were identified based on the HMM profile of the MYB domain by using HMMER software (http://hmmer.org/download.html). The SMART website (http://smart.embl-heidelberg.de), the HMMER website (https://www.ebi.ac.uk/Tools/hmmer/), and Batch CD-Search in NCBI database (https://www.ncbi.nlm.nih.gov/Structure/bwrpsb/bwrpsb.cgi) were utilized to confirm all the TaMYBs containing two or three MYB repeats.

### Chromosomal distribution, gene duplication, and synteny analysis

The position information of identified *TaMYBs* was acquired by blasting the MYB sequences with wheat genome database (IWGSC RefSeq v1.0). Based on the position information, a physical map was drawn with MapInspect software (https://mapinspect.software.informer.com/). The analysis of duplication and synteny relationship was performed by local blast program. Then synteny relationship map was drawn with TB tools [55]. The identification criterion of the tandem duplication events with a small modification was as following: 1) alignment length is over 80% of the full length of the gene, 2) aligned region has over 80% identity, 3) no genes are inserted between them, 4) the E-value < e-10 [56, 57]. Segmental duplication events respectively detected in subgenome A/B/D are defined as following: 1) alignment length is longer than 600 KB, and 2) aligned region has over 90% identity [57, 58]. Ka/Ks was calculated with TB tools software [55].

### Phylogenetic analysis and gene characterization analysis

The TaMYBs protein sequences of wheat were aligned using the software ClustalX [59]. Based on the multiple sequence alignment results, phylogenetic tree was generated by using MEGA 7 software coupled with Neighbor-Joining method with a bootstrap of 1000 replicates [60]. CDS and genomic sequences were downloaded from IWGSC RefSeq v1.0 (https://wheat-urgi.versailles.inra.fr/Seq-Repository/Assemblies), then exon/intron structures of *TaMYBs* were obtained by using online software Gene Structure Display Server 2.0 (http://gsds.cbi.pku.edu.cn/). The pI and Mw were predicted with the ExPASy-Compute pI/Mw tool (https://web.expasy.org/compute_pi/). The subcelluar location information was predicted by utilizing online software WOLF PSORT (https://wolfpsort.hgc.jp/).

### Expression profiles analysis based on RNA-seq data

The RNA-seq data titled “choulet_URGI” were downloaded to analyze the spatial and temporal expression profiles of *TaMYBs* in wheat from expVIP website (http://www.wheat-expression.com/). For expression profile analysis of *TaMYBs* under different stress (cold, heat and drought, powdery mildew pathogen and stripe rust pathogen, and phosphate starvation), the RNA-seq data titled “SRP043554”, “SRP045409”, “SRP041017”, and “DRP000768” were obtained from expVIP website. The TPM values were normalized with Z-score method, as follow: Z sample-i = [(log_2_(Signal sample-i)-mean(log_2_(Signal) of all samples)]/[Standard deviation (log_2_(Signal) of all samples)]. The up- or down-regulated genes under stress treatments were screened with strict conditions as following: 1) the expression level was up or down 2-fold regulation, i.e. log_2_ fold change (FC) > 1 or < − 1, 2) *P* value < 0.05. Then heatmaps and volcano plots were drawn by using R program.

### Plant material and expression analysis experiment

Wheat (*T.aestivum* cv. Chinese Spring) was used as the plant material in this study, and the seeds were acquired from the Genetic Engineering International Cooperation Base of Chinese Ministry of Science and Technology, College of Life Science and Technology, Huazhong University of Science and Technology (HUST). Seeds were germinated in the dark and were cultivated in a greenhouse (12 h light/12 h dark cycle at 22 °C). For drought and salt stress treatments, 14-day-old seedlings were cultured in solutions containing 20% PEG6000 (w/v) or 200 mM NaCl for 24 h. All samples were collected at the time points (0 h, 1 h, 3 h, 6 h, 12 h, 24 h), frozen in liquid nitrogen, and then stored at − 80 °C for subsequent RNA extraction. Total RNA was extracted from different samples with a Plant Total RNA Extraction Kit (Zoman, Beijing, China). First-strand cDNA was synthesized with the FastQuant RT Kit (TIANGEN, Beijing, China). qRT-PCR was performed with SuperReal PreMix Plus Kits (TIANGEN, Beijing, China) on the machine CFX Connect Real-Time System (Bio-Rad, Hercules, CA, United States). Expression data was analyzed with the comparative 2(T)(−Delta Delta C) method [61]. The primers used in this assay are listed in Additional file 1: Table S8. The housekeeping wheat gene actin (accession no. AB181991.1) was used as the internal control.

### Plant transformation

Tobacco (*Nicotiana tabacum*) was used as the plant material in this study, and the seeds were acquired from the Genetic Engineering International Cooperation Base of Chinese Ministry of Science and Technology, College of Life Science and Technology, Huazhong University of Science and Technology (HUST). To generate transgenic tobacco plants that overexpressed *TaMYB344*, the ORF containing the terminator codon was cloned into the pBI121 vector under the control of *cauliflower mosaic virus 35S* promoter with X*ba*I/*Bam*HI restriction sites. The pBI121-TaMYB344-GFP and pBI121-GFP vectors were transformed into *Agrobacterium tumefaciens* strain EHA105 respectively. Tobacco plant transformation was accomplished using the *A. tumefaciens*-mediated leaf disk method [62]. Three independent transgenic T_2_ lines were obtained in which the expression level of *TaMYB344* was examined by qRT-PCR.

### Stress tolerance analysis of the transgenic plants

The stress tolerance of WT, VC, and OE lines was analyzed. Seeds were surface sterilized with 75% ethanol for 1 min and 10% H_2_O_2_ for 8 min. For heat stress treatment, the seeds were placed in 50 °C chamber for 60 min. Then the seeds were sown on 1/2 MS medium and incubated in a growth chamber (12 h light/12 h dark cycle at 22 °C). After 4 days, the germination rates were counted. To analyze the drought and salt stress tolerance of transgenic plants, 2-week-old seedlings were planted in pots and grown in the greenhouse under a 12 h light/12 h dark cycle at 22 °C. For each biological replicate, about 50 tobacco plants (10 pots) of each line were treated. For drought stress tolerance assay of transgenic plants, 4-week-old plants were withheld water for 16 days and then re-watered for 1 week. For the salt stress tolerance assay of transgenic plants, 3-week-old plants grown in pots were treated with 500 mM NaCl for 20 days in a container. Supplemental NaCl solution was added to the container every 3 days throughout the treatment period. The stomatal aperture assay was accomplished in accordance with slight modifications: the duration of dehydration treatment was modified to 40 min [63]. The results were photographed by microscopy (IX71, Olympus, Japan).

### Subcellular localization and transcriptional activation analysis

An expression vector pMD18-ubi-GFP was constructed with the maize ubiquitin promoter and *GFP* gene to perform transient expression experiment. Then, the ORF of *TaMYB344* was amplified using specific primers containing *Hin*dIII/*Spe*I restriction sites (Additional file 1: Table S8) and cloned into pMD18-ubi-GFP vector fusing with the 5′-terminal of the *GFP* gene. The obtained recombinant vector ubiqutin:: TaMYB344-GFP and the control vector pMD18-Ubi-GFP were respectively transformed into onion epidermal cells via particle bombardment. The results were observed and photographed with fluorescence microscopy (IX71, Olympus, Japan).

The Clontech Matchmaker™ Yeast One-Hybrid system (TBUSA, CA, USA), a GAL4-based yeast one-hybrid system, was employed to examine the transactivation activity. The complete ORF as well as various truncated ORFs of *TaMYB344* were amplified by PCR using specific primers containing *Eco*RI/*Bam*HI restriction sites (Additional file 1: Table S8). These fragments were then inserted into the pGBKT7 vector to construct corresponding recombinant. Vector pGBKT7 was used as the negative control plasmid. All these plasmids were transformed into the yeast strain AH109, respectively. Yeast transformation and screening were performed in accordance with the users’ manual (Clontech, United States).

### Statistical analysis

Statistical analysis was performed with Perseus software [64] and Student’s *t*-test.

## Supplementary Information

**Additional file 1: **Sequences of MYBs in wheat (**Table S1**); Characteristic features of MYBs in wheat (**Table S2**); Syntenic relationships of MYBs in wheat (**Table S3**); The Ka/Ks of duplication gene pairs (**Table S4**); The expression data of MYBs in wheat (**Table S5**); The up/down regulated MYBs in wheat under various stress treatment (**Table S6**); Putative *cis*-acting regulatory elements of *TaMYB344* promoter sequence (**Table S7**); Primer pairs for amplification (**Table S8**).

**Additional file 2: **The gene structure of *TaMYBs* in wheat (**Figure S1–4**); Phylogenetic tree of R2R3-MYBs in wheat (**Figure S5**); The expression patterns of *R1R2R3-MYBs* in wheat (**Figure S6)**; The variance of expression level of *R2R3-MYBs* (**Figure S7–9**); The overexpressing transgenic lines of *TaMYB344* (**Figure S10**).

## Data Availability

The sequencing data for the genomics sequences is available in the URGI (https://urgi.versailles.inra.fr/download/iwgsc/IWGSC_RefSeq_Assemblies/v1.0/). The public RNA-seq data are available on expVIP website (http://www.wheat-expression.com/).

## Abbreviations

HMM: Hidden Markov model
CDS: Coding sequences
TPM: The transcript per million
WT: Wild type
VC: Vacant control
ORF: Open reading frames
X-α-gal: X-α-galactoside
IWGSC: The International Wheat Genome Sequencing Consortium
pI: Isoelectric point
Mw: Molecular weight
qRT-PCR: Quantitative real-time PCR
