# Supplementary material for: Genome-wide identification of R2R3-MYB family in wheat and functional characteristics of the abiotic stress responsive gene TaMYB344
Source: BMC Genomics. 2020 Nov 12;21:792. doi: 10.1186/s12864-020-07175-9 (PMC7659103; doi:10.1186/s12864-020-07175-9)
Supplement: Supplementary file 2 — Additional file 2: The gene structure of TaMYBs in wheat (Figure S1–4); Phylogenetic tree of R2R3-MYBs in wheat (Figure S5); The expression patterns of R1R2R3-MYBs in wheat (Figure S6); The variance of expression level of R2R3-MYBs (Figure S7–9); The overexpressing transgenic lines of TaMYB344 (Figure S10). [file 12864_2020_7175_MOESM2_ESM.zip › Additional file 2-revise.pdf]

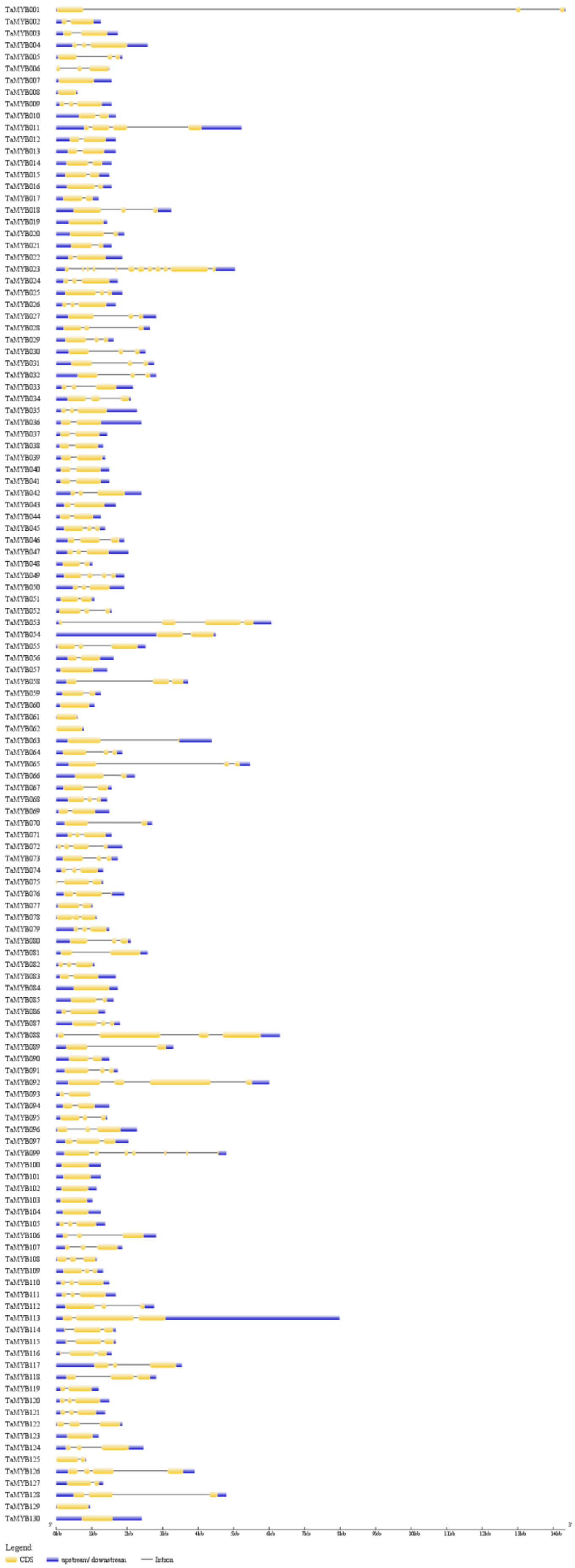
TaMYB001
TaMYB002
TaMYB003
TaMYB004
TaMYB005
TaMYB006
TaMYB007
TaMYB008
TaMYB009
TaMYB010
TaMYB011
TaMYB012
TaMYB013
TaMYB014
TaMYB015
TaMYB016
TaMYB017
TaMYB018
TaMYB019
TaMYB020
TaMYB021
TaMYB022
TaMYB023
TaMYB024
TaMYB025
TaMYB026
TaMYB027
TaMYB028
TaMYB029
TaMYB030
TaMYB031
TaMYB032
TaMYB033
TaMYB034
TaMYB035
TaMYB036
TaMYB037
TaMYB038
TaMYB039
TaMYB040
TaMYB041
TaMYB042
TaMYB043
TaMYB044
TaMYB045
TaMYB046
TaMYB047
TaMYB048
TaMYB049
TaMYB050
TaMYB051
TaMYB052
TaMYB053
TaMYB054
TaMYB055
TaMYB056
TaMYB057
TaMYB058
TaMYB059
TaMYB060
TaMYB061
TaMYB062
TaMYB063
TaMYB064
TaMYB065
TaMYB066
TaMYB067
TaMYB068
TaMYB069
TaMYB070
TaMYB071
TaMYB072
TaMYB073
TaMYB074
TaMYB075
TaMYB076
TaMYB077
TaMYB078
TaMYB079
TaMYB080
TaMYB081
TaMYB082
TaMYB083
TaMYB084
TaMYB085
TaMYB086
TaMYB087
TaMYB088
TaMYB089
TaMYB090
TaMYB091
TaMYB092
TaMYB093
TaMYB094
TaMYB095
TaMYB096
TaMYB097
TaMYB099
TaMYB100
TaMYB101
TaMYB102
TaMYB103
TaMYB104
TaMYB105
TaMYB106
TaMYB107
TaMYB108
TaMYB109
TaMYB110
TaMYB111
TaMYB112
TaMYB113
TaMYB114
TaMYB115
TaMYB116
TaMYB117
TaMYB118
TaMYB119
TaMYB120
TaMYB121
TaMYB122
TaMYB123
TaMYB124
TaMYB125
TaMYB126
TaMYB127
TaMYB128
TaMYB129
TaMYB130
5'
3'
0kb
1kb
2kb
3kb
4kb
5kb
6kb
7kb
8kb
9kb
10kb
11kb
12kb
13kb
14kb
Legend
CDS
upstream/ downstream
Intron

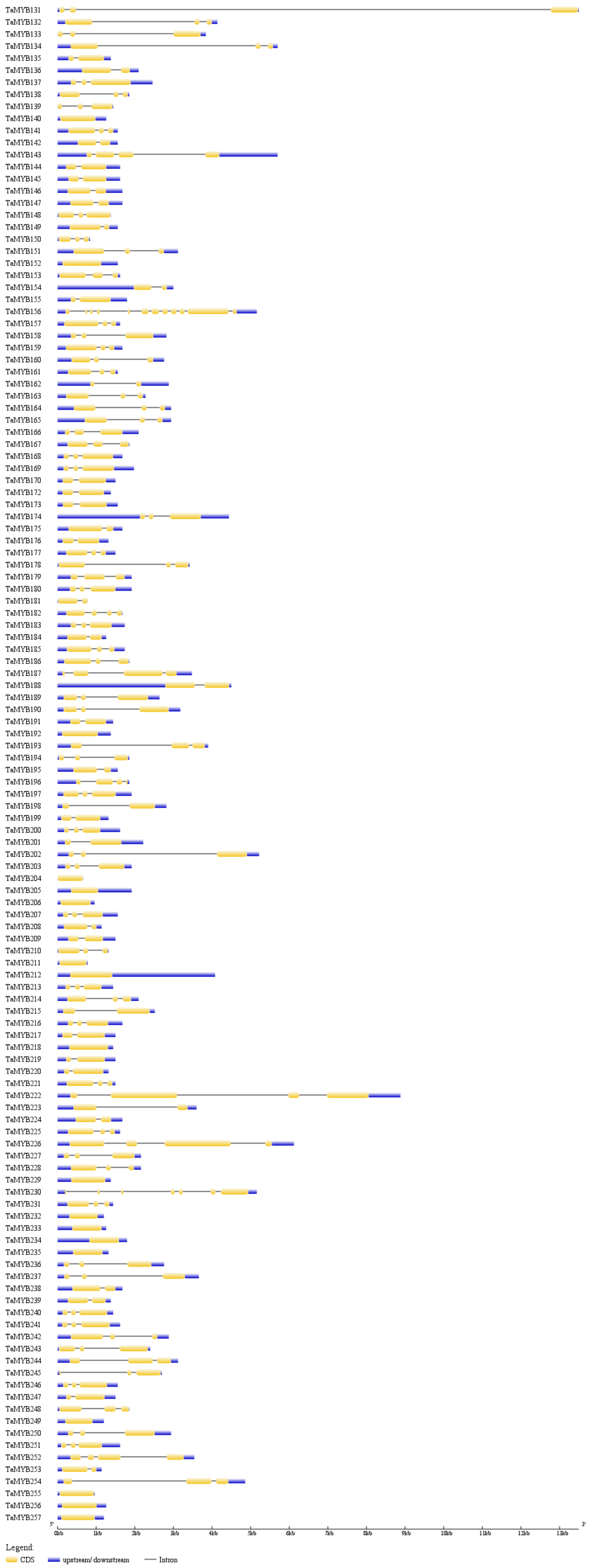
TaMYB131
TaMYB132
TaMYB133
TaMYB134
TaMYB135
TaMYB136
TaMYB137
TaMYB138
TaMYB139
TaMYB140
TaMYB141
TaMYB142
TaMYB143
TaMYB144
TaMYB145
TaMYB146
TaMYB147
TaMYB148
TaMYB149
TaMYB150
TaMYB151
TaMYB152
TaMYB153
TaMYB154
TaMYB155
TaMYB156
TaMYB157
TaMYB158
TaMYB159
TaMYB160
TaMYB161
TaMYB162
TaMYB163
TaMYB164
TaMYB165
TaMYB166
TaMYB167
TaMYB168
TaMYB169
TaMYB170
TaMYB172
TaMYB173
TaMYB174
TaMYB175
TaMYB176
TaMYB177
TaMYB178
TaMYB179
TaMYB180
TaMYB181
TaMYB182
TaMYB183
TaMYB184
TaMYB185
TaMYB186
TaMYB187
TaMYB188
TaMYB189
TaMYB190
TaMYB191
TaMYB192
TaMYB193
TaMYB194
TaMYB195
TaMYB196
TaMYB197
TaMYB198
TaMYB199
TaMYB200
TaMYB201
TaMYB202
TaMYB203
TaMYB204
TaMYB205
TaMYB206
TaMYB207
TaMYB208
TaMYB209
TaMYB210
TaMYB211
TaMYB212
TaMYB213
TaMYB214
TaMYB215
TaMYB216
TaMYB217
TaMYB218
TaMYB219
TaMYB220
TaMYB221
TaMYB222
TaMYB223
TaMYB224
TaMYB225
TaMYB226
TaMYB227
TaMYB228
TaMYB229
TaMYB230
TaMYB231
TaMYB232
TaMYB233
TaMYB234
TaMYB235
TaMYB236
TaMYB237
TaMYB238
TaMYB239
TaMYB240
TaMYB241
TaMYB242
TaMYB243
TaMYB244
TaMYB245
TaMYB246
TaMYB247
TaMYB248
TaMYB249
TaMYB250
TaMYB251
TaMYB252
TaMYB253
TaMYB254
TaMYB255
TaMYB256
TaMYB257
5'
0kb
1kb
2kb
3kb
4kb
5kb
6kb
7kb
8kb
9kb
10kb
11kb
12kb
13kb
3'
Legend:
CDS
upstream/downstream
Intron

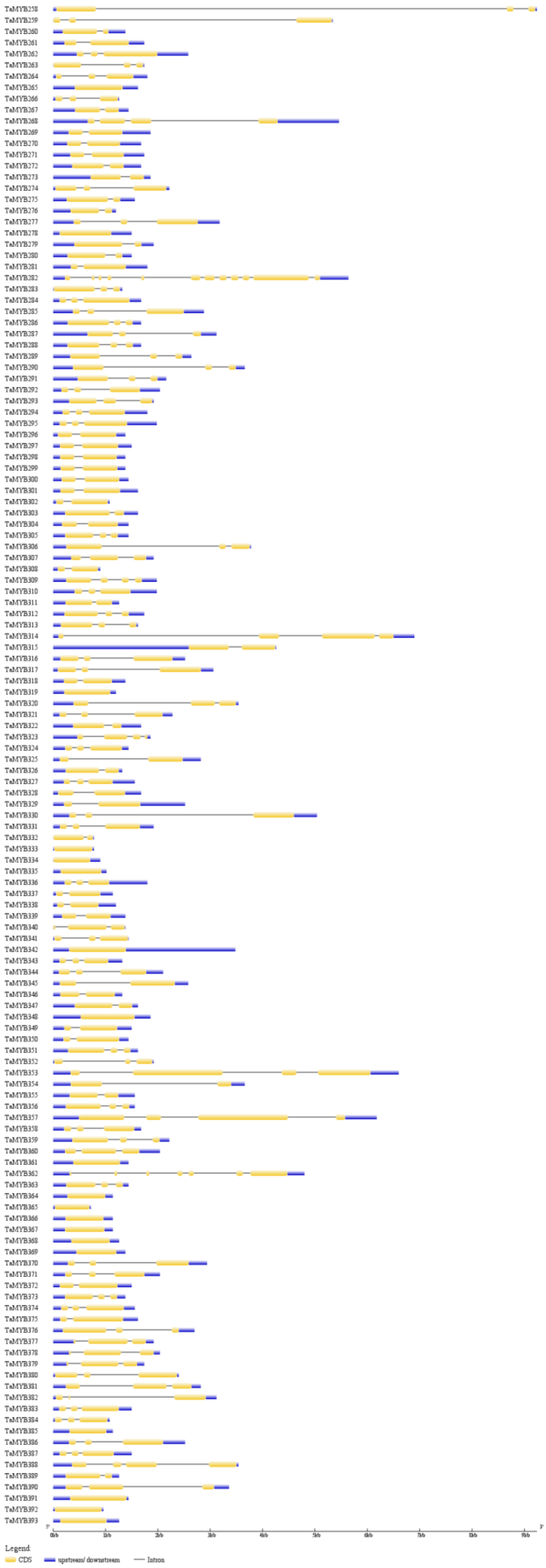
TaMYB258
TaMYB259
TaMYB260
TaMYB261
TaMYB262
TaMYB263
TaMYB264
TaMYB265
TaMYB266
TaMYB267
TaMYB268
TaMYB269
TaMYB270
TaMYB271
TaMYB272
TaMYB273
TaMYB274
TaMYB275
TaMYB276
TaMYB277
TaMYB278
TaMYB279
TaMYB280
TaMYB281
TaMYB282
TaMYB283
TaMYB284
TaMYB285
TaMYB286
TaMYB287
TaMYB288
TaMYB289
TaMYB290
TaMYB291
TaMYB292
TaMYB293
TaMYB294
TaMYB295
TaMYB296
TaMYB297
TaMYB298
TaMYB299
TaMYB300
TaMYB301
TaMYB302
TaMYB303
TaMYB304
TaMYB305
TaMYB306
TaMYB307
TaMYB308
TaMYB309
TaMYB310
TaMYB311
TaMYB312
TaMYB313
TaMYB314
TaMYB315
TaMYB316
TaMYB317
TaMYB318
TaMYB319
TaMYB320
TaMYB321
TaMYB322
TaMYB323
TaMYB324
TaMYB325
TaMYB326
TaMYB327
TaMYB328
TaMYB329
TaMYB330
TaMYB331
TaMYB332
TaMYB333
TaMYB334
TaMYB335
TaMYB336
TaMYB337
TaMYB338
TaMYB339
TaMYB340
TaMYB341
TaMYB342
TaMYB343
TaMYB344
TaMYB345
TaMYB346
TaMYB347
TaMYB348
TaMYB349
TaMYB350
TaMYB351
TaMYB352
TaMYB353
TaMYB354
TaMYB355
TaMYB356
TaMYB357
TaMYB358
TaMYB359
TaMYB360
TaMYB361
TaMYB362
TaMYB363
TaMYB364
TaMYB365
TaMYB366
TaMYB367
TaMYB368
TaMYB369
TaMYB370
TaMYB371
TaMYB372
TaMYB373
TaMYB374
TaMYB375
TaMYB376
TaMYB377
TaMYB378
TaMYB379
TaMYB380
TaMYB381
TaMYB382
TaMYB383
TaMYB384
TaMYB385
TaMYB386
TaMYB387
TaMYB388
TaMYB389
TaMYB390
TaMYB391
TaMYB392
TaMYB393
5'
3'
0kb
1kb
2kb
3kb
4kb
5kb
6kb
7kb
8kb
9kb
Legend:
CDS
upstream/ downstream
Intron

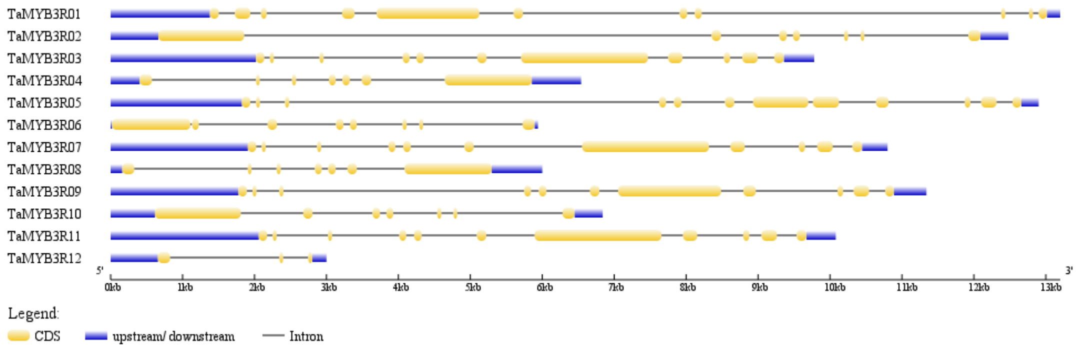

Figure.S1-4 The gene structure of *TaMYB* genes in wheat.

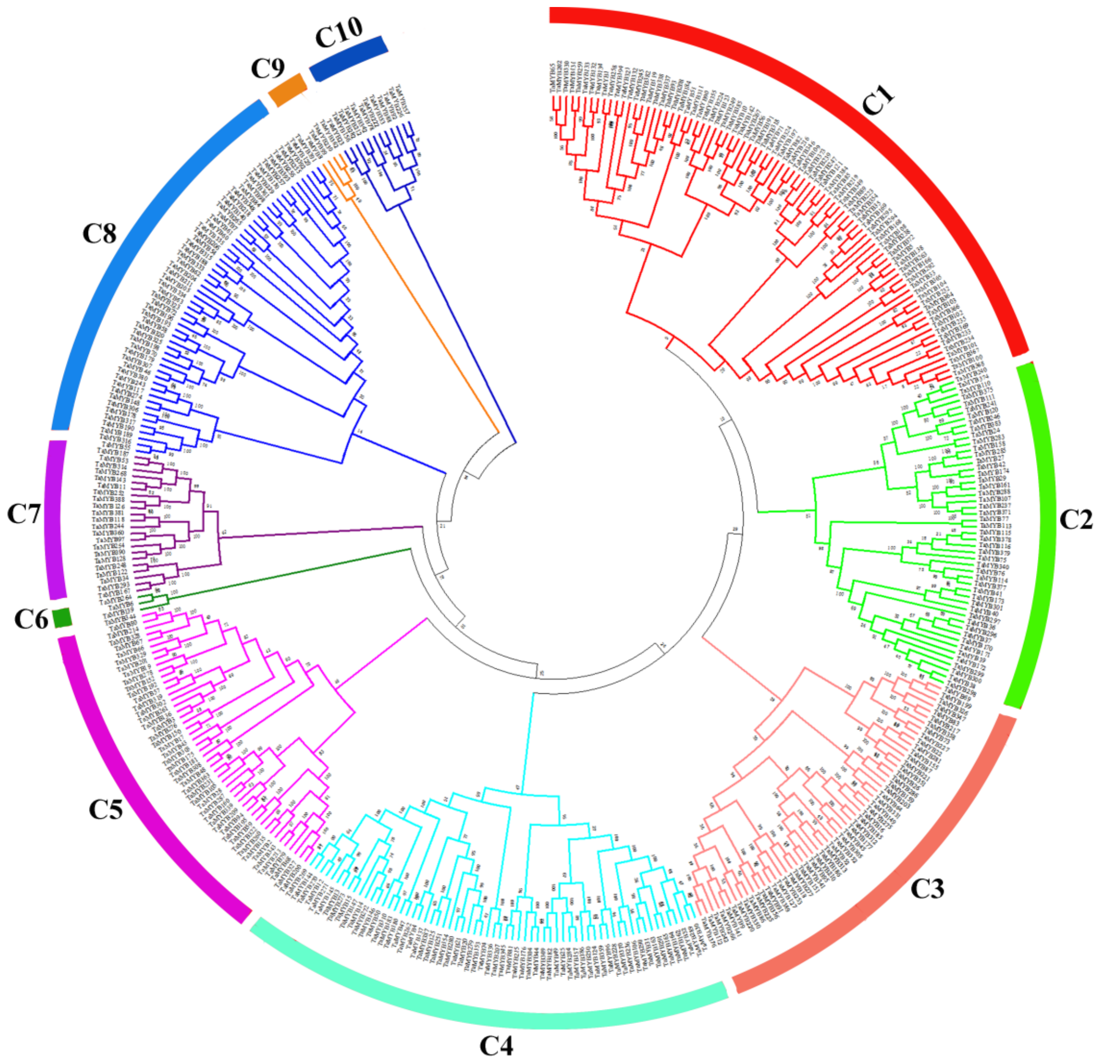

Figure.S5 Phylogenetic tree of 393 R2R3-MYBs in wheat. All R2R3-MYBs were divided into 10 clusters (C1-C10). The picture was generated by using MEGA 7 software coupled with Neighbor-Joining method with a bootstrap of 1000 replicates.

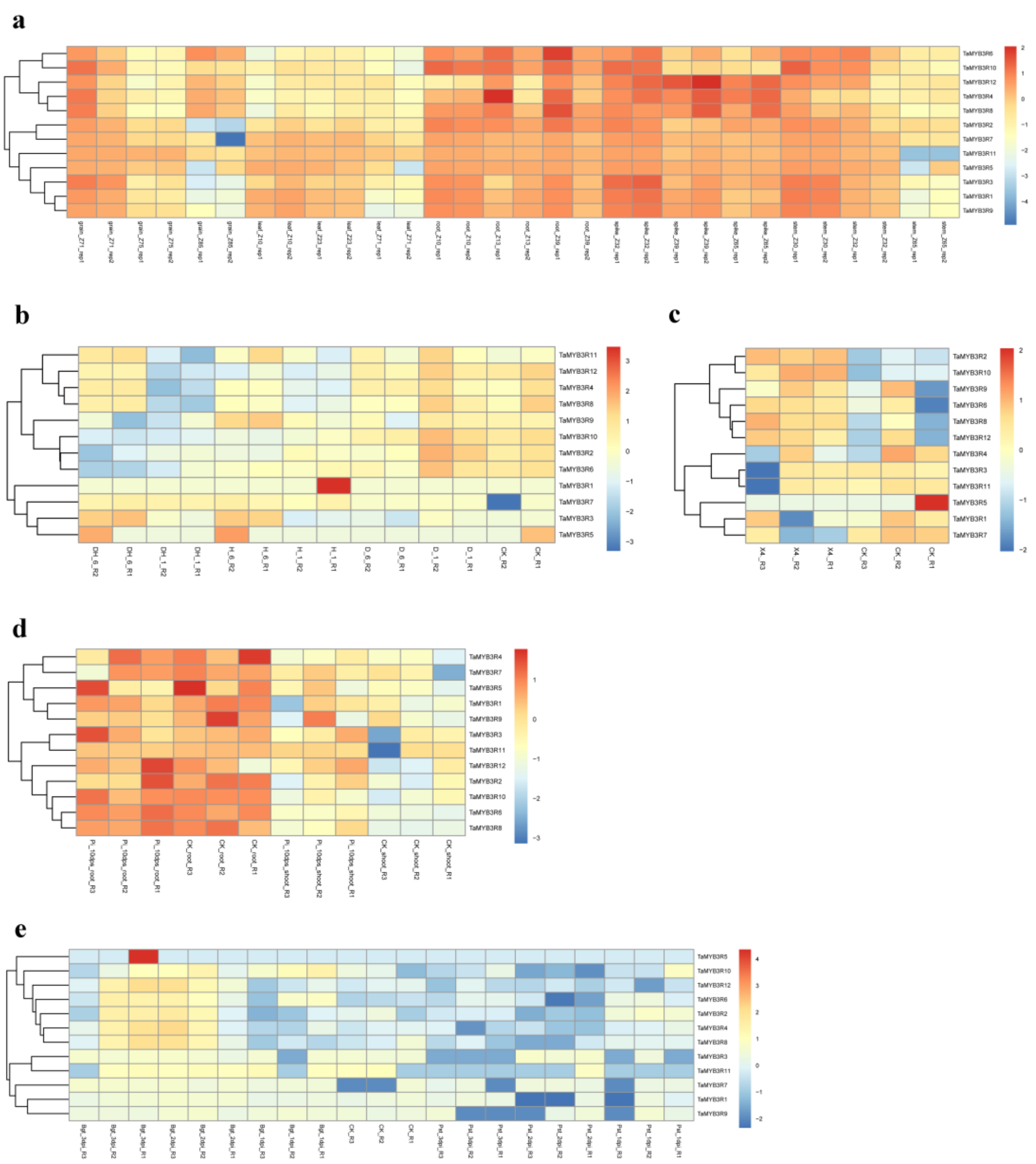

Figure.S6 The expression patterns of *R1R2R3-MYBs* in wheat. (a) The expression patterns of *R1R2R3-MYBs* in different tissues. Row coordinate meanings refer to Figure.4 caption. (b) The expression patterns of *R1R2R3-MYBs* under drought/heat. Row coordinate meanings refer to Figure.5 caption. (c) The expression patterns of *R1R2R3-MYBs* under cold stress. X4 means low temperature 4 °C; CK means room temperature 23°C. (d) The expression patterns of *R1R2R3-MYBs* under Pi-starvation environment. Pi_10dps_root: the root of wheat under 10 days post Pi-starvation stress; Pi_10dps_shoot: the shoot of wheat under 10 days post Pi-starvation stress. (e) The expression patterns of *R1R2R3-MYBs* under diseases. Bgt_1, _2, and _3dpi: 1, 2, and 3

days post Powdery mildew pathogen E09 infection, respectively; Pst_1, _2, and _3dpi: 1, 2, and 3 days post Stripe rust pathogen CYR31 infection, respectively. Heatmap was created by R program based on the data of the transcript per million (TPM) values which were normalized by Z-score method. The red and blue cells respectively represent highest and lowest expression level.

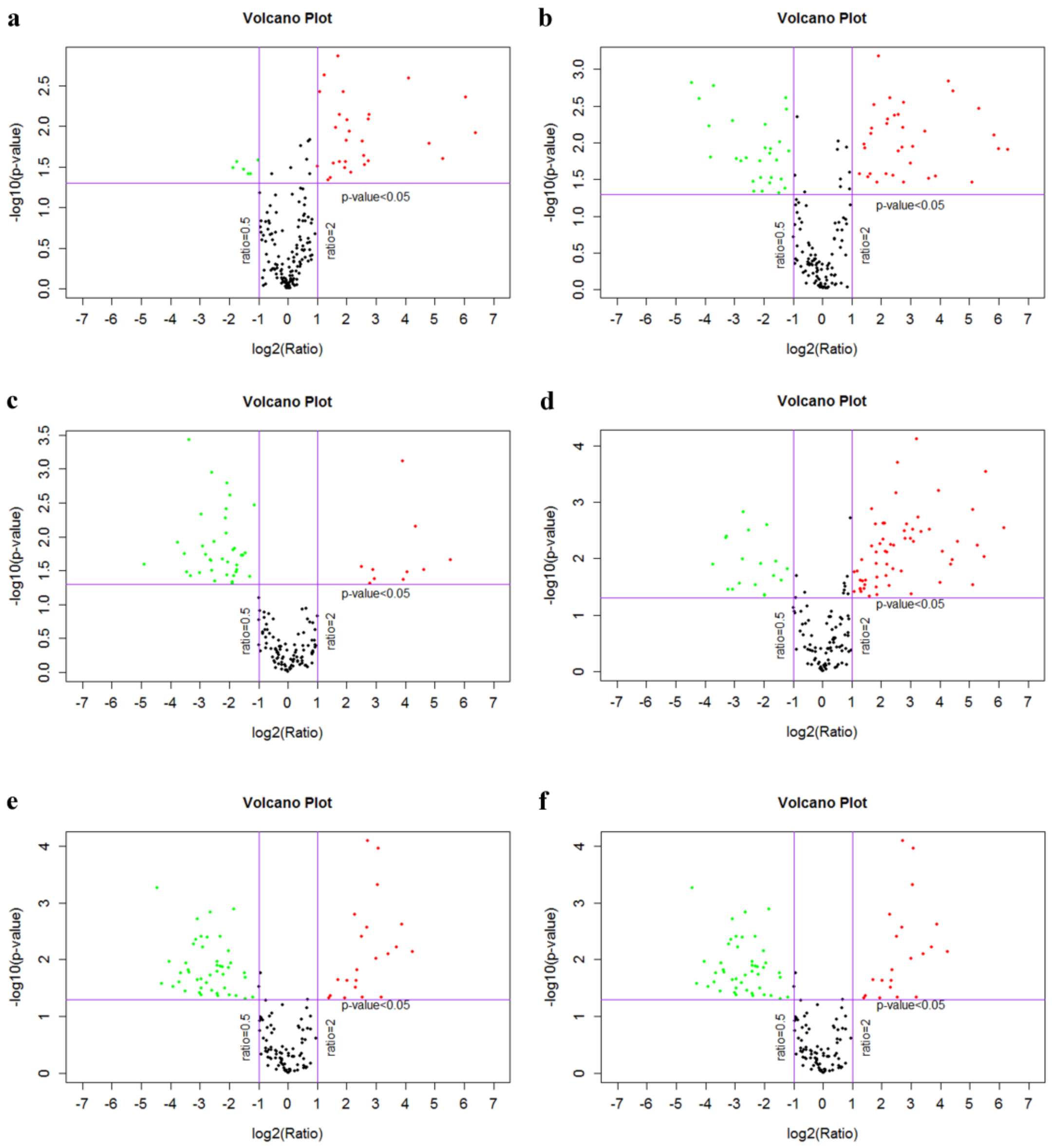

Figure.S7 The variance of expression level of *TaR2R3-MYBs*. (a, b) under drought stress after 1 and 6 hours. (c, d) under heat stress after 1 and 6 hours. (e, f) under drought and heat stress after 1 and 6 hours. The volcano plots were drawn by R program. The red, green, and black dots respectively show up-regulated, down-regulated, and no

change expression level of *R2R3-MYB* genes. Strict screening conditions as following: 1) the expression levels were up or down 2-folds regulation, i.e. ratio (FC) < 0.5 or ratio (FC) > 2; 2) *P* value < 0.05.

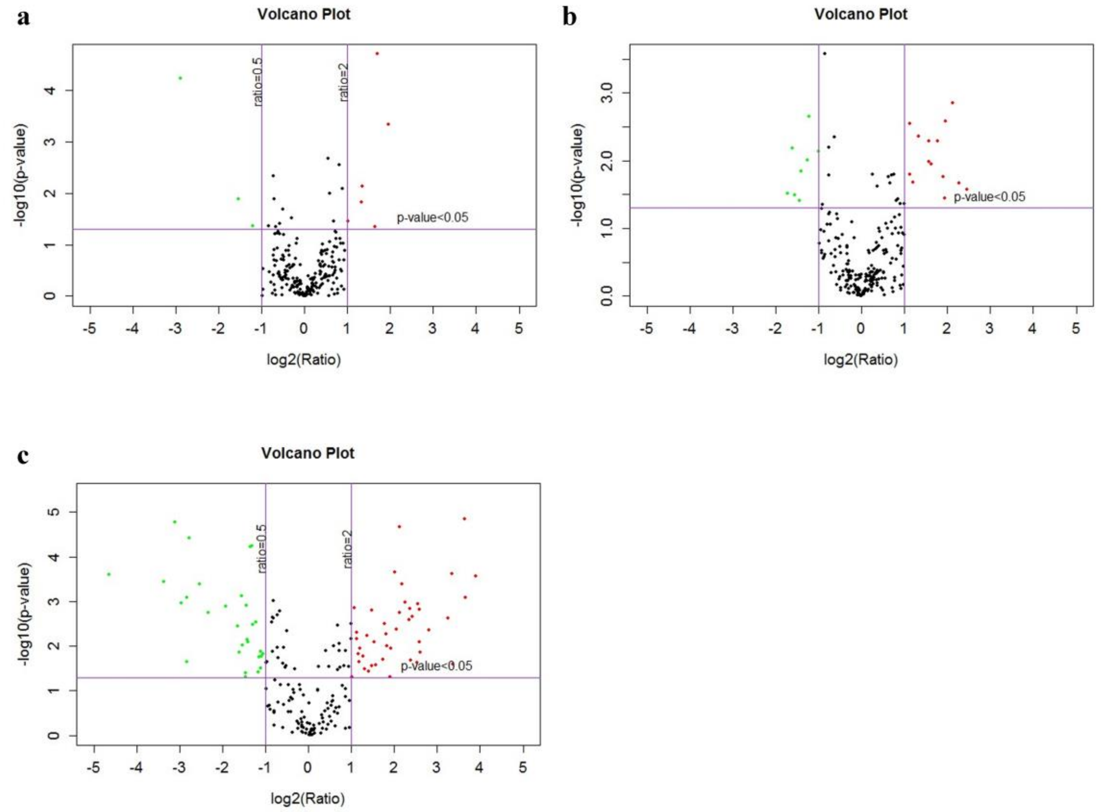

Figure.S8 The variance of expression level of *TaR2R3-MYBs*. (a, b) 10 days post Pi-starvation stress of root and shoot of wheat. (c) under cold (4 °C) treatment. The volcano plots were drawn by R program. The red, green, and black dots respectively show up-regulated, down-regulated, and no change expression level of *R2R3-MYB* genes. Strict screening conditions as following: 1) the expression levels were up or down 2-folds regulation, i.e. ratio (FC) < 0.5 or ratio (FC) > 2; 2) *P* value < 0.05.

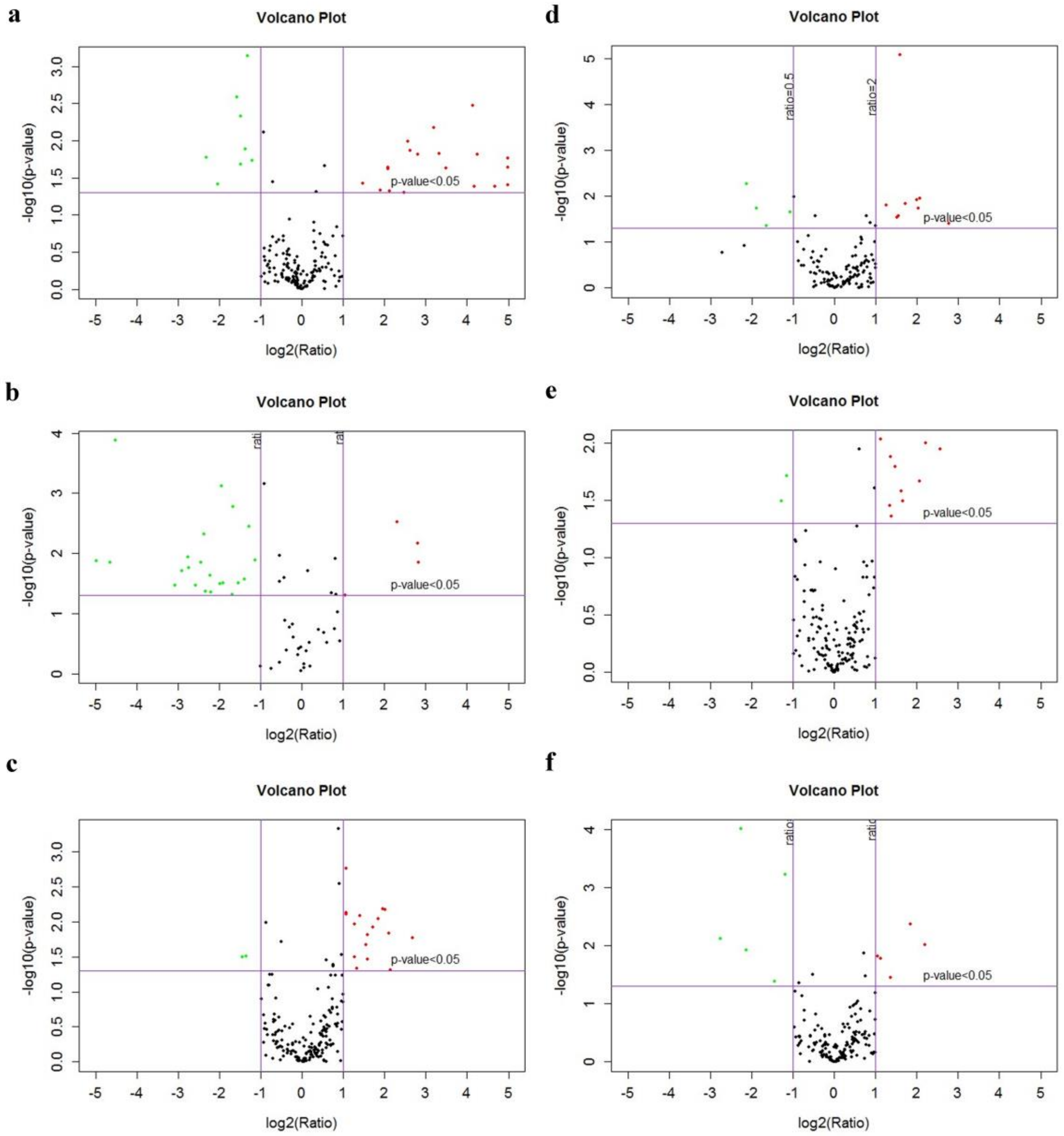

Figure.S9 The variance of expression level of *TaR2R3-MYBs*. (a, b, c) 1, 2, and 3 days post Powdery mildew pathogen E09 infection, respectively; (d, e, f) 1, 2, and 3 days post Stripe rust pathogen CYR31 infection, respectively. The volcano plots were drawn by R program. The red, green, and black dots respectively show up-regulated, down-regulated, and no change expression level of *R2R3-MYB* genes. Strict screening conditions as following: 1) the expression levels were up or down 2-folds regulation, i.e. ratio (FC) < 0.5 or ratio (FC) > 2; 2) *P* value < 0.05.

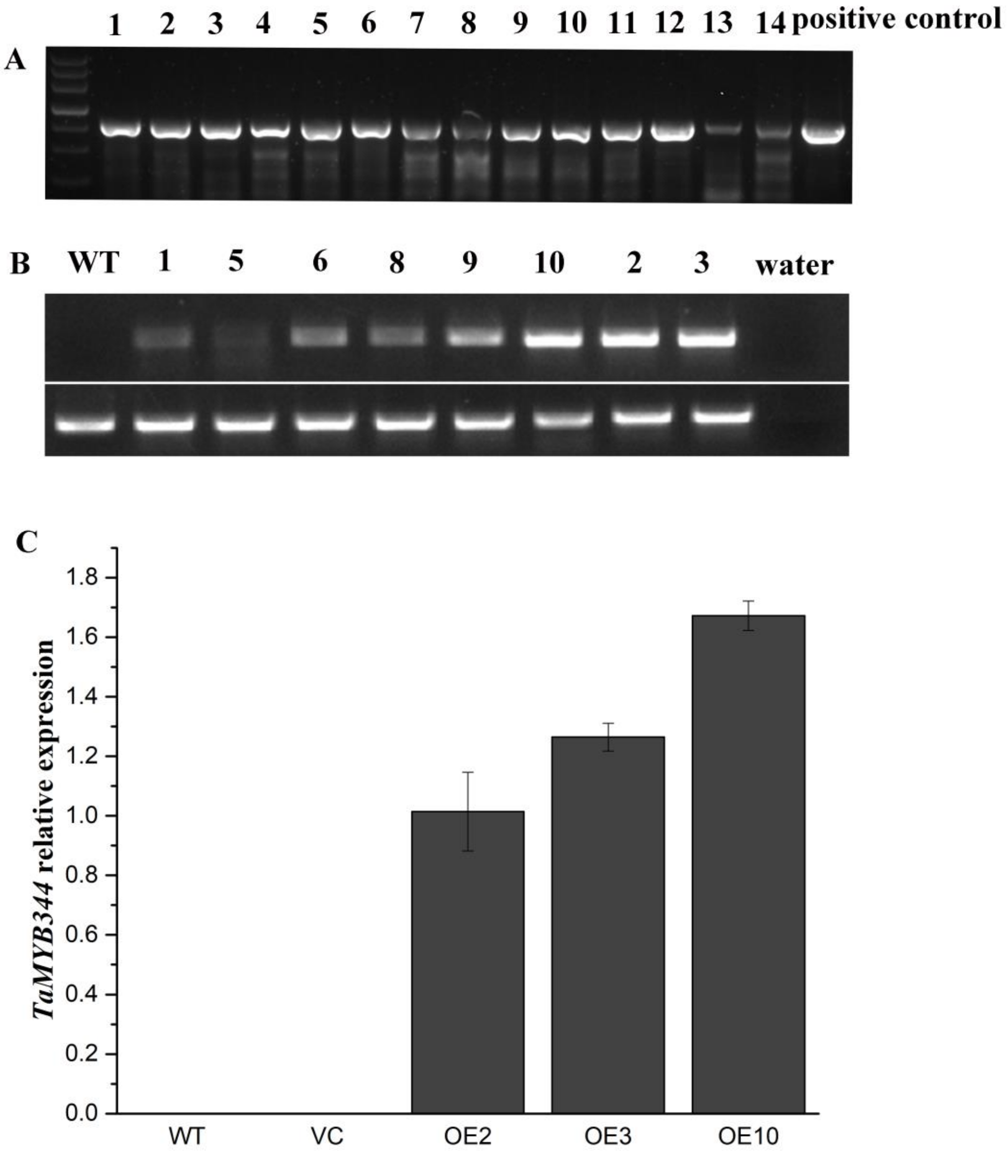

Figure.S10 The overexpressing transgenic lines of *TaMYB344*. (a) Identification of positive lines at DNA level by PCR. The relative expression level of *TaMYB344* in WT, VC, and transgenic lines determined by semi-quantitative PCR (b) and qRT-PCR (c).
