# Supplementary material for: Genome-wide identification of R2R3-MYB family in wheat and functional characteristics of the abiotic stress responsive gene TaMYB344
Source: BMC Genomics. 2020 Nov 12;21:792. doi: 10.1186/s12864-020-07175-9 (PMC7659103; doi:10.1186/s12864-020-07175-9)
Supplement: Supplementary file 2 — Additional file 2: The gene structure of TaMYBs in wheat (Figure S1–4); Phylogenetic tree of R2R3-MYBs in wheat (Figure S5); The expression patterns of R1R2R3-MYBs in wheat (Figure S6); The variance of expression level of R2R3-MYBs (Figure S7–9); The overexpressing transgenic lines of TaMYB344 (Figure S10). [file 12864_2020_7175_MOESM2_ESM.zip › Original gel images .docx]

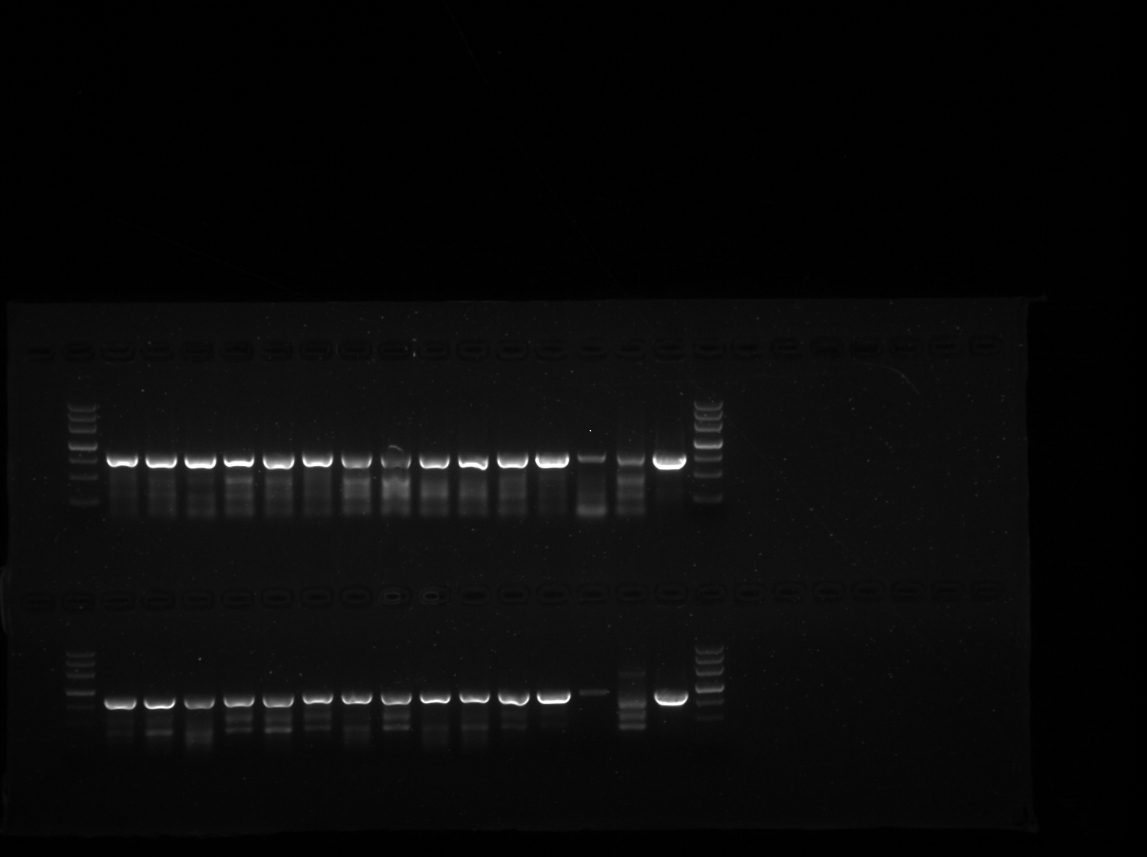


1 2 3 4 5 6 7 8 9 10 11 12 13 14 positive control

800bp

Figure.S10A The original gel image of identification of positive *TaMYB344* overexpressing lines at DNA level by PCR. The numbers indicate the different lines.


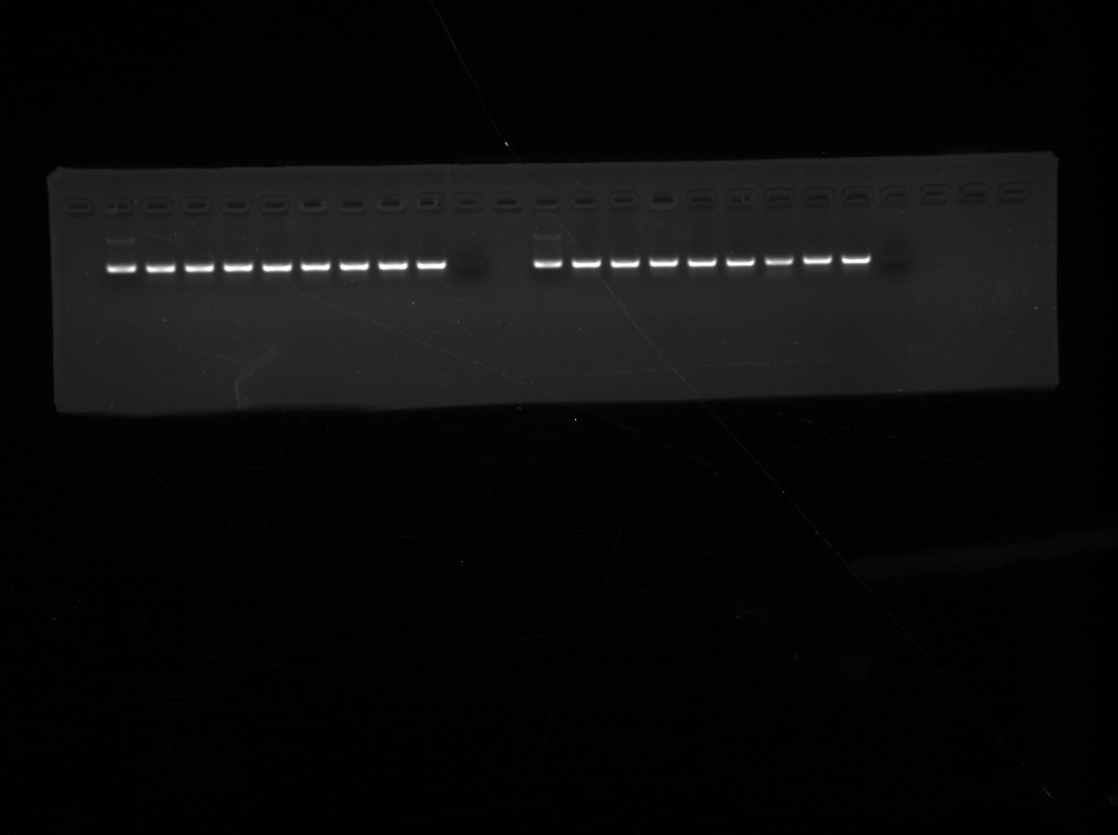


(*NtUbiqutin*)

WT 1 5 6 8 9 10 2 3 water


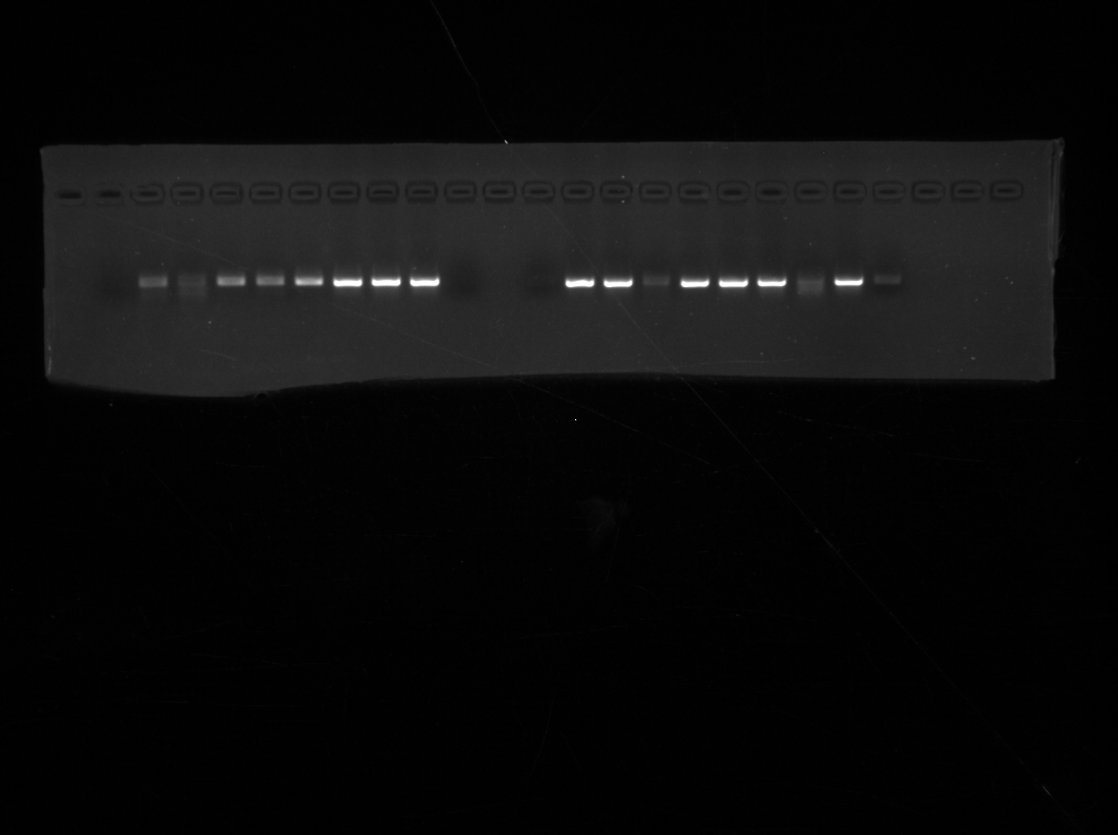


(*TaMYB344*)

WT 1 5 6 8 9 10 2 3 water

Figure.S10B The original gel image of the relative expression level of *TaMYB344* in WT, VC, and transgenic lines determined by semi-quantitative PCR. The numbers indicate the different lines.
